# An epigenome atlas of mouse adipocytes

**DOI:** 10.1016/j.molmet.2025.102197

**Published:** 2025-06-27

**Authors:** Laura C. Hinte, Adhideb Ghosh, Daniel Castellano-Castillo, Christian Wolfrum, Ferdinand von Meyenn

**Affiliations:** 1 Laboratory of Nutrition and Metabolic Epigenetics, Institute of Food, Nutrition and Health, Department of Health Sciences and Technology, ETH Zurich, Switzerland; 2 Laboratory of Translational Nutrition Biology, Institute of Food, Nutrition and Health, Department of Health Sciences and Technology, ETH Zurich, Switzerland

**Keywords:** Adipocytes, Epigenetics, Enhancers, Beige, White, Brown, Histone marks, Multiomics

## Abstract

**Objective:**

Epigenetic modifications including histone post translational modifications can influence gene expression in adipocytes, potentially contributing to metabolic dysfunctions, obesity, and insulin resistance. Despite recent advances in the characterization of the mouse adipocyte epigenome, epigenetic characterization of adipocytes *in vivo* has been challenging, particularly across different adipose depots and of several epigenetic modifications.

**Methods:**

Here, we use specific reporter mice labelling brown, beige and white adipocytes, diphtheria toxin-mediated ablation of beige adipocytes, and Cleavage Under Targets and Tagmentation (CUT&Tag) to generate paired single mouse datasets of five histone marks. We perform an integrative multi-omics factor analysis (MOFA) of H3K4me3, H3K27me3, H3K4me1, H3K27ac and H3K9me3 in brown, white and beige adipocytes from three distinct mouse adipose tissue depots obtained during cold exposure and thermoneutrality.

**Results:**

Our analysis reveals that enhancers distinguish adipocytes by their tissue of origin, with H3K4me1 deposition differentiating between beige and brown adipocytes. Beige adipocytes poised promoters associated to thermogenic genes during warming. Diphtheria toxin-mediated ablation of beige adipocytes shows that non-beigeing white adipocytes in inguinal adipose tissue and beige adipocytes are not inherently epigenetically different suggesting that they share a common developmental progenitor.

**Conclusions:**

These paired multimodal data comprise an extensive resource (https://github.com/vonMeyennLab/mAT_CE_Atlas) for the further exploration of the mouse adipocyte epigenome which will enable discovery of regulatory elements governing adipocyte identity and gene regulation.

## Highlights

• Epigenome atlas of five histone marks from mouse brown, beige and white adipocytes.
• Putative enhancers identified by ChromHMM dinstinguish adipocytes by tissue of origin.
• Beige adipocytes retain a thermogenic memory and poise thermogenic genes during warming.
• Beiged (Ucp1+) and non-beiged (Ucp1-) inguinal adipocytes are epigenetically similar.

## 1 Introduction

Epigenetic mechanisms and chromatin modifications are essential for development, differentiation and identity maintenance of brown, beige, and white adipocytes *in vitro* and *in vivo* [[1], [2], [3], [4], [5], [6], [7], [8]]. So far, most datasets on histone post-translational modifications (hPTMs), chromatin accessibility, and DNA methylation were generated from bulk adipose tissue (AT) samples or from *in vitro* cultured mesenchymal stem cell or cell line derived adipocytes. Epigenetic datasets from mouse adipocytes *in vivo* in the context of obesity [6,9] or cold exposure [7] have been generated using the sophisticated NuTRAP [10] mouse model. The latter datasets are still limited to at most two hPTMs from separate samples from pooled tissues, thus a comprehensive characterization of the mouse adipocyte epigenome from lean mice remains incomplete. To address this gap, we generated and analysed paired *in vivo* datasets of five key hPTMs relevant for gene regulation and identity maintenance, providing a comprehensive characterization of the mouse adipocyte epigenome.

### 1.1 Integrative multimodal atlas of the mouse adipocyte epigenome

**Figure 1:**
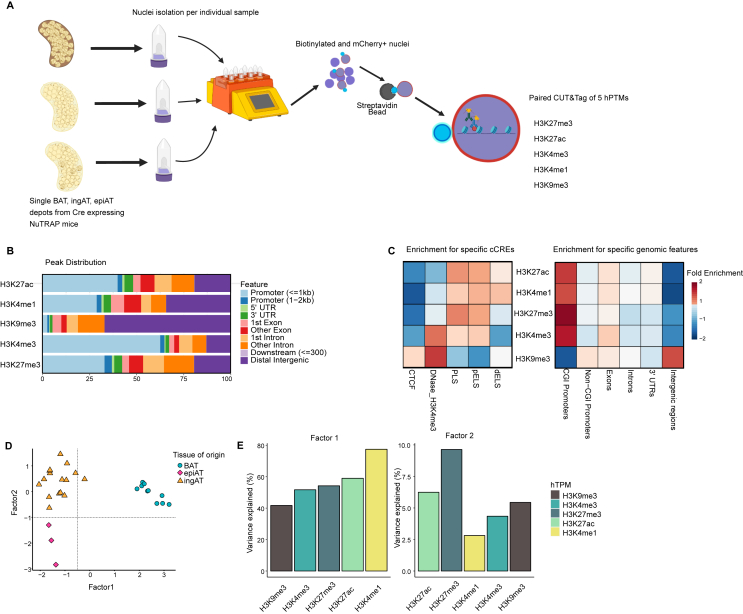
**Integrative multimodal analysis of the mouse adipocyte epigenome.** (A) Workflow of paired CUT&Tag from labelled adipocytes of one adipose tissue depot from BAT, ingAT or epiAT. AdipoCre x NuTRAP or Ucp1ERCre x NuTRAP mice are used. Nuclei are isolated from frozen tissue, pulled down using streptavidin coated beads and subjected to parallel CUT&Tag assaying five hPTMs. Figure was created with BioRender.com. (B) Distribution of called peaks from all samples per hPTM across genomic features in % of peaks falling into a region. (C) Peak fold enrichment of called peaks from each representative CUT&Tag libraries for ENCODE candidate cis-regulatory elements (cCREs) with promoter like signatures (PLs), proximal enhancer like signatures (pELS) or distal enhancer like signatures (dELS), scaled from −2 to 2 (left) and peak fold enrichment of called peaks from each CUT&Tag library for genomic features, scaled from −2 to 2 (right). (D) Multi-Omics Factor Analysis (MOFA) factor plot showing the clustering along factors 1, 2 of labelled adipocytes from BAT, ingAT and epiAT. Associations from Factor 1 (*P* = 1.16 × 10^−16^) and Factor 2 (*P* = 7.43 × 10^−7^) to tissue of origin are significant. Each dot corresponds to one biological replicate. For each replicate all 5 hPTMs are represented in one dot. epiAT *n* = 3, BAT *n* = 10, ingAT *n* = 15. (E) Percentage of variance explained by MOFA factor 1 and 2 across one of 5 modalities. Significance of associations of MOFA factor scores to covariates was tested using fitted ANOVAs using Benjamini-Hochberg procedure for correction for multiple testing.

We isolated adipocyte nuclei from brown adipose tissue (BAT), inguinal adipose tissue (ingAT), and epididymal adipose tissue (epiAT) of AdipoCre x NuTRAP and Ucp1ERCre x NuTRAP mice housed under different conditions and generated Cleavage under Targets and Tagmentation (CUT&Tag) [11] datasets for five hPTMs (Figure 1A). We assayed H3K4me3, marking active promoters and transcriptional start sites [12,13], H3K4me1, enriched at active and primed enhancers and often used as an enhancer marker [14], H3K27ac, indicative of active enhancers and promoters [[15], [16], [17]], H3K27me3, a repressive mark deposited by polycomb repressive complex 2 associated with facultative heterochromatin [18] but also repressed [19,20] and poised or bivalent promoters [21], and H3K9me3, which is enriched in constitutive heterochromatin [22] and often colocalized with DNA methylation [23] (Figures S1A-B, S2). The observed genomic localisation corresponds to the expected genomic loci (Figure 1B–C). We then performed Multi-Omics Factor Analysis (MOFA) [[24], [25], [26]] with the paired datasets to investigate the sources of variability in our data. MOFA uses multivariate dimension reduction to identify source of variation shared across multiple datasets and represent them in terms of common latent factors. MOFA identified one factor, factor 1 (*P* = 1.16 × 10^−16^) (Figure 1D, Fig. S3), that significantly separated adipocytes derived from BAT and white adipose tissues (WAT). H3K4me1 plays a significant role in this separation (Figure 1E, Fig. S3) suggesting that enhancers, which are crucial for defining cellular identity and fate and represent cell lineages [27,28], distinguish adipocytes derived from BAT and WAT, rather than promoter usage or repressed chromatin regions.

### 1.2 Brown and beige adipocytes have distinct enhancers

**Figure 2:**
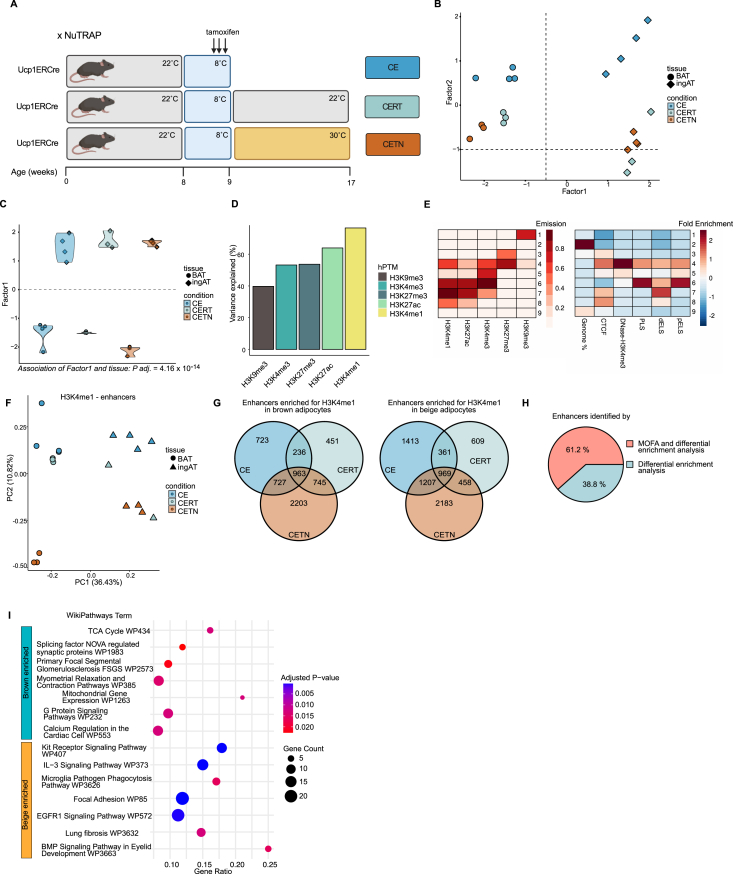
**Enhancers distinguish brown from beige adipocytes.** (A) Experimental setup of the cold exposure and recovery study. Ucp1ERCre x NuTRAP mice are cold exposed for 1 week during which tamoxifen is administered. Subsequently, mice are either sacrificed (CE) or allowed to recover at 22 °C (CERT) or 30 °C (CETN) before tissue harvest. Figure was created with BioRender.com and modified thereafter. (B) MOFA factor plot showing the clustering along factors 1, 2 of labelled beige and brown adipocytes from BAT and ingAT. Association of Factor 1 to tissue of origin is significant (*P* = 4.16 × 10^−14^). Each dot corresponds to one biological replicate. For each replicate all 5 hPTMs are represented in one dot. (C) MOFA violin plot displaying separation of adipocyte samples along factor 1. Each dot corresponds to one biological replicate. For each replicate all 5 hPTMs are represented in one dot. (D) Percentage of variance explained by MOFA factor 1 across 5 hPTMs. (E) ChromHMM analysis representation of the adipocyte hPTM profiles for Ucp1ERCre adipocytes (left) by nine chromatin states. The colour scale corresponds to the emission parameter of each hPTM for each state. And fold enrichment of ChromHMM states for total genomic coverage and ENCODE cCREs scaled from −2 to 2 (right). States 7 and 8 are identified as enhancers. (F) Principal Component Analysis (PCA) plot of H3K4me1 quantified in beige and brown adipocytes specific enhancers from E. Each dot corresponds to an individual biological replicate. (G) Overlap of enhancers identified by differential enrichment analysis of H3K4me1 in enhancers from e in beige and brown adipocytes from the same condition (CE, CERT, CETN) for brown (left) and beige (right) adipocytes. (H) Percentage of specific enhancers identified in g that are also contributing to the variance explained by H3K4me1 in MOFA factor 1 from c. (I) Top (significant) pathway terms for genes linked to brown (top) and beige (bottom) adipocyte specific enhancers from G based on the Wikipathways database. CE BAT *n* = 4, CE ingAT *n* = 4, CERT BAT *n* = 3, CERT ingAT *n* = 3, CETN BAT *n* = 3, CETN ingAT *n* = 3. Fisher’s exact test with Benjamini-Hochberg method for correction of multiple testing was used for GSEA (I). Significance of associations of MOFA factor scores to variables was tested using fitted ANOVAs using Benjamini-Hochberg procedure for correction for multiple testing (C). (For interpretation of the references to colour in this figure legend, the reader is referred to the Web version of this article.)

Given our observation that H3K4me1, an enhancer mark, accounts for nearly 80% of the variability in our paired datasets, primarily distinguishing BAT-derived from WAT-derived adipocytes, we decided to explore the epigenetic differences between beige and brown adipocytes, which are phenotypically similar but originate from different AT depots. To this end we collected inguinal AT (ingAT) and BAT from Ucp1ERCre x NuTRAP mice where we had labelled UCP1 expressing cells using tamoxifen administration during 1 week of cold (8 °C) exposure. We collected samples directly after cold exposure (CE), or after 8 weeks of recovery at 22 °C (CERT), or 30 °C (CETN) (Figure 2A). We then isolated the labelled beige and brown adipocytes from these samples. By applying MOFA to these paired datasets of 5 hPTMs for each sample, we observed a strong separation by MOFA factor 1 of beige and brown adipocytes (Figure 2B–C, Fig. S4A) (*P* = 4.16 × 10^−14^). Factor 1 was driven mainly by H3K4me1 (75 % of variance explained) and H3K27ac (63 % of variance explained) confirming that beige and brown adipocytes used very different enhancer sets (Figure 2D, Fig. S4A). Motivated by these results, we used ChromHMM [29] to compile beige and brown specific enhancer sets. ChromHMM is based on a multivariate hidden Markov model and integrates multiple datasets to discover the major re-occurring combinatorial and spatial patterns in the genome, thereby allowing to identify chromatin “states” which are enriched for specific hPTMs. We identified enhancers by selecting states that were enriched for H3K4me1 and H3K27ac but not H3K4me3, and were annotated in the ENCODE [30] database as candidate cis-regulatory elements (cCREs) with distal enhancer like signatures (dELS) (Figure 2E). Using these enhancer annotations, we performed differential enrichment analysis of H3K4me1 in beige and brown adipocytes for each condition to identify beige and brown specific enhancers. We found a strong separation of brown and beige adipocytes (Figure 2F) with several thousands of enhancers being differentially enriched. Corroborating Roh et al. [7], the comparison of enhancers in beige and brown adipocytes of CETN mice yielded the most differentially marked enhancers (Figure 2G). To define beige and brown adipocyte specific enhancer sets we intersected enhancers that were enriched in brown and/or beige adipocytes of CETN, CERT and CE mice (Figure 2G). We obtained 963 enhancers that were brown adipocyte specific across all conditions and 969 enhancers that were beige specific (Figure 2G, Tables S1 and S2). Of note, >60 % of these enhancers had also been identified by MOFA factor 1 (Figure 2H) as features explaining the variability between beige and brown adipocytes.

H3K4me1 marking is present on both poised and active enhancers, offering insights into potential cellular function or cell identity, but not indicating whether an enhancer is active. To assess the status of beige and/or brown specific enhancers and their putatively linked genes (see Methods for details), we investigated their H3K27ac status. We found that most beige and brown specific enhancers and genes linked to them were H3K27ac marked (Fig. S4B-C) at 8 °C, 22 °C, and 30 °C and thus likely active. Wondering, which biological pathways might be regulated by active enhancers that distinguish brown from beige adipocytes, we annotated enhancers to their closest gene and performed gene set enrichment analysis (GSEA). Active brown enhancers were related to mitochondrial respiration and myometrial pathways (Figure 2I), while active beige enhancers were linked to genes related to EGFR signalling and fibrosis/extra cellular matrix (ECM) remodelling (Figure 2I). Overall, enhancers and to a lesser extent active promoters, differentiate brown from beige adipocytes, supporting other studies that these cell types are distinct from each other and have different cell identities and developmental origin [31].

### 1.3 Beige adipocytes poise thermogenic genes upon rewarming

**Figure 3:**
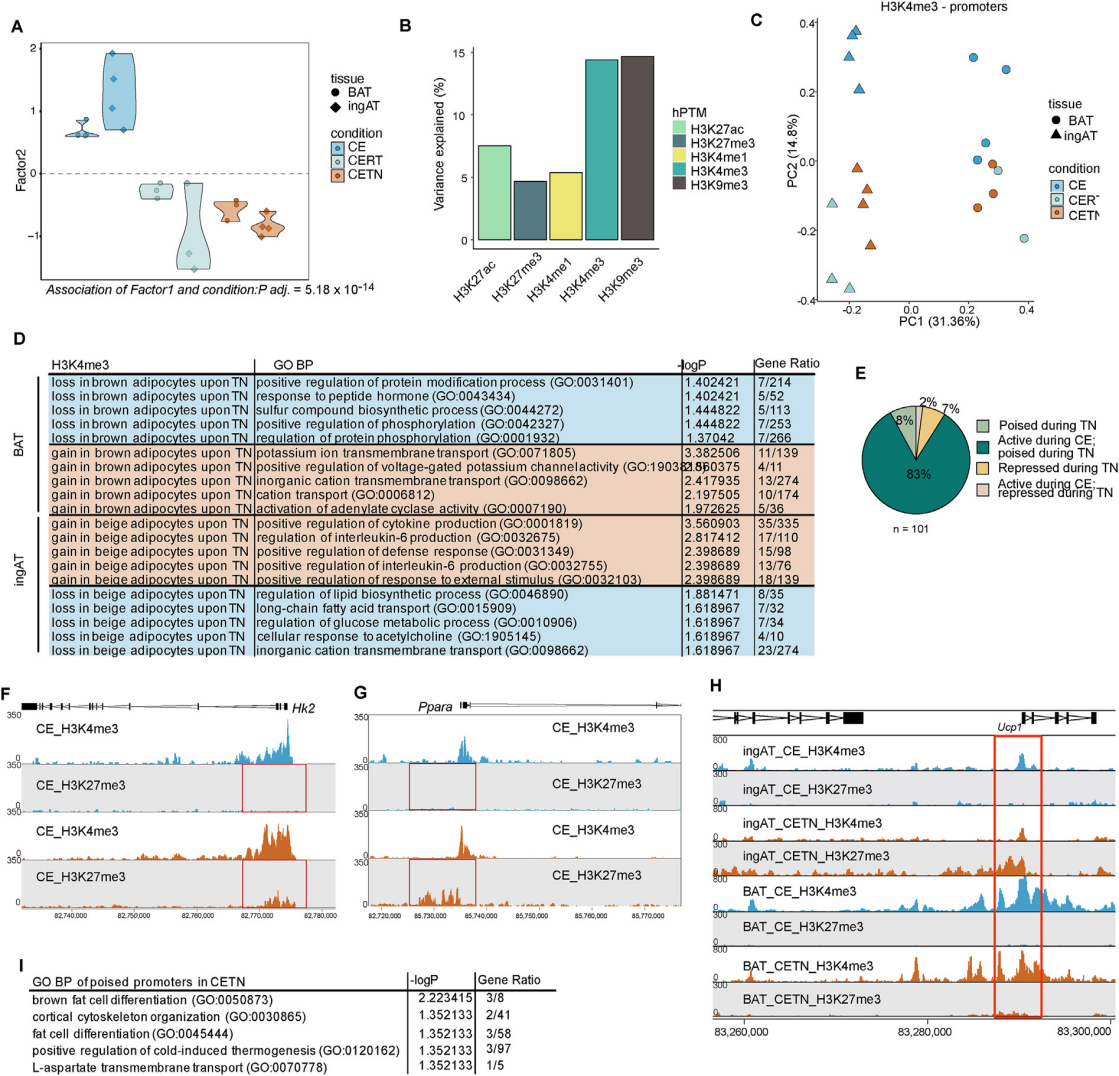
**Beige adipocytes poise thermogenic genes upon rewarming.** (A) MOFA violin plot displaying separation of beige and brown adipocyte samples from ingAT and BAT along factor 2. Association of Factor 2 to condition is significant (*P* = 5.18 × 10^−6^). Each dot corresponds to one biological replicate. For each replicate all 5 hPTMs are represented in one dot. (B) Percentage of variance explained by MOFA factor 2 from 2c across 5 hPTMs. (C) PCA plot of H3K4me3 quantified in promoters of beige and brown adipocyte samples. Each dot corresponds to an individual biological replicate. (D) Top (significant) terms and -log of adjusted p-value for genes linked to promoters gaining H3K4me3 in CETN or losing H3K4me3 enrichment in brown adipocytes from BAT (top) and beige adipocytes from ingAT (bottom) based on GO Biological Process terms. (E) Pie chart depicting the poising status of promoters enriched for H3K27me3 in CETN beige adipocytes across conditions. (F) Genome browser track of the *Hk2* promoter of H3K4me3 and H3K27me3 signals of CE and CETN beige adipocytes. (G) Genome browser track of the *Ppara* promoter of H3K4me3 and H3K27me3 signals of CE and CETN beige adipocytes. hPTM signals have a common scale. (H) Genome browser track of the *Ucp1* promoter of H3K4me3 and H3K27me3 signals of CE and CETN beige adipocytes from ingAT and brown adipocytes from BAT in CE and CETN. (I) Top (significant) terms and -log of adjusted p-value for genes linked to poised promoters of beige adipocytes in CETN based on GO Biological Process terms. CE BAT *n* = 4, CE ingAT *n* = 4, CERT BAT *n* = 3, CERT ingAT *n* = 3, CETN BAT *n* = 3, CETN ingAT *n* = 3. Fisher’s exact test with Benjamini-Hochberg method for correction of multiple testing was used for GSEA (D, I). Significance of associations of MOFA factor scores to variables was tested using fitted ANOVAs using Benjamini-Hochberg procedure for correction for multiple testing (A). (For interpretation of the references to colour in this figure legend, the reader is referred to the Web version of this article.)

Beige adipocytes have been shown to lose H3K27ac at specific loci in a temperature-dependent manner following rewarming [7]. Expanding on this, we questioned whether, in addition to enhancer usage, other epigenetic mechanisms are responsive to cold exposure. Exploring our MOFA results, we found that beige and brown adipocytes from cold exposed mice were significantly separated from those of other conditions (*P* = 5.18 × 10^−6^), by factor 2 (Figure 3A, Fig. S4A). Factor 2 is mostly driven by H3K9me3 and H3K4me3 (Figure 3B).

H3K9me3 is a hPTM associated with constitutive heterochromatin and is almost absent in gene bodies or promoters (Figure 1B), limiting its annotation to genes and GSEA. Differential enrichment analysis of peaks revealed that H3K9me3 was affected to a greater extend by CE and rewarming in brown adipocytes than in beige adipocytes (Fig. S4D). We found 194 regions that were cold-specific repressed and 52 warm-specific repressed by H3K9me3 in both beige and brown adipocytes (Fig. S4D, Table S3). These regions accounted for only 6 % of the H3K9me3 differentially enriched regions after warming, indicating that although H3K9me3 deposition is remodelled upon CE and rewarming in both beige and brown adipocytes, this remodelling does not follow a common pattern.

Roh et al. [7], and our own analyses show a loss of active H3K27ac at thermogenic enhancers and gene bodies in beige, but not brown, adipocytes upon rewarming. Given that MOFA also indicated H3K4me3 as a contributing modality to Factor 2, we wondered whether promoters would display a similar dynamic. We performed a differential enrichment analysis for H3K4me3 at promoters between brown and beige adipocytes in cold and warm conditions respectively. Principal component analysis (PCA) of H3K4me3 at promoters separated CE from CETN and CERT adipocytes (Figure 3C) along PC2. Interestingly, in beige adipocytes, the gain of H3K4me3 during rewarming is linked to genes involved in the response to and production of IL-6. In contrast, brown adipocytes gain H3K4me3 deposition at genes related to potassium membrane transport (Figure 3D). Further, beige adipocytes in warm conditions show a loss of H3K4me3 at genes involved in glucose and fatty acid metabolism and response to acetylcholine, processes associated with thermogenesis. In warmed brown adipocytes, there is a relative H3K4me3 loss at genes related to protein modification and phosphorylation (Figure 3D). Notably, the active hPTM H3K4me3 was preserved at the *Ucp1* gene – a key marker gene for thermogenesis [32] – in both beige and brown adipocytes during warming.

CETN beige adipocytes have been shown to not express *Ucp1* and lose H3K27ac at enhancers related to thermogenic genes [7], thus we wondered if such genes were poised for future activation in beige adipocytes by co-deposition of H3K4me3 and H3K27me3 at their promoters. Indeed, differential analysis of H3K27me3 and H3K4me3 deposition at corresponding promoters in CE and CETN beige adipocytes revealed, that during warming promoters became poised rather than repressed. While only 101 promoters gained H3K27me3 robustly during warming (Figure 3E, Table S4), promoters of genes with major relevance for thermogenic, such *Ppara*, *Hk2, and Ucp1* were amongst these promoters being poised by co-deposition of H3K4me3 and H3K27me3 (Figure 3F–H). GSEA revealed that poised promoters were strongly associated to thermogenesis and brown fat cell differentiation (Figure 3I).

### 1.4 Beige adipocytes are not distinct from white adipocytes in ingAT

**Figure 4:**
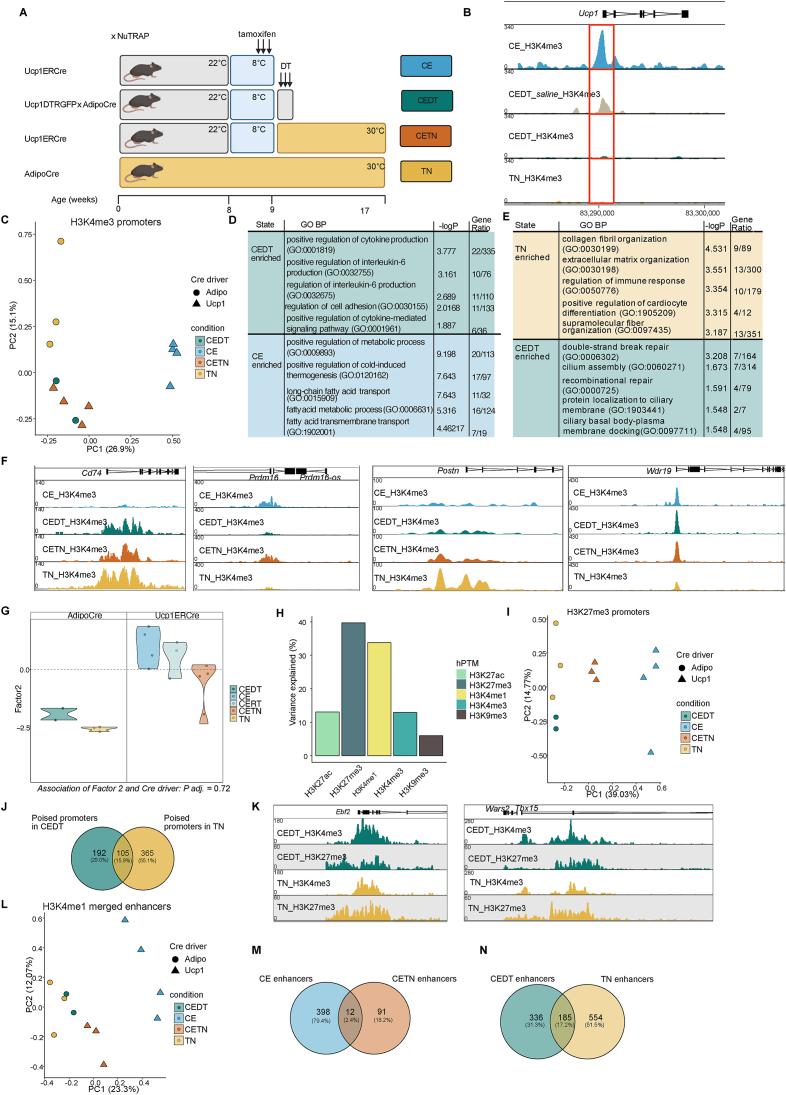
**Beige adipocytes are not distinct from white adipocytes in ingAT.** (A) Experimental setup of the cold exposure and Ucp1+ cells ablation study. Ucp1ERCre x NuTRAP mice are cold exposed for 1 week during which tamoxifen is administered. Subsequently, mice are either sacrificed (CE) or allowed to recover at 30 °C (CETN) before tissue harvest. Ucp1DTRGFP x AdipoCre x NuTRAP (CEDT) mice are cold exposed and injected with diphtheria toxin after cold exposure before tissue harvest. AdipoCre x NuTRAP mice are housed at 30 C (TN) since birth. Figure was created with BioRender.com and modified thereafter. (A) Genome browser track of the *Ucp1* promoter region with scaled H3K4me3 signal of CE beige adipocytes, adipocytes from CEDT injected with saline instead of diphtheria toxin (CEDT_*saline*), CEDT adipocytes and TN adipocytes. (C) PCA plot of H3K4me3 quantified in promoters of CE, CETN, CEDT and TN adipocyte samples. Each dot corresponds to an individual biological replicate. (D) Top (significant) terms and -log of adjusted p-value for genes linked to promoters enriched in (non-beige) CEDT adipocytes (top) and (beige) CE (bottom) adipocytes based on GO Biological Process terms. € Top (significant) terms and -log of adjusted p-value for genes linked to promoters enriched in TN adipocytes (top) and CEDT (bottom) adipocytes based on GO Biological Process terms. (F) Genome browser tracks of scaled H3K4me3 signals at promoters of *Cd74*, *Prdm16*, *Postn* and *Wdr19* of CE, CEDT, CETN and TN adipocytes. (G) MOFA violin plot displaying separation of adipocyte samples along Factor 2. The association of the Factor 2 to the Cre driver is not significant. Each dot corresponds to one biological replicate. For each replicate all 5 hPTMs are represented in one dot. (H) Percentage of variance explained by MOFA factor 2 across 5 hPTMs. (I) PCA plot of H3K27me3 quantified in promoters of CE, CETN, CEDT and TN adipocyte samples. Each dot corresponds to an individual biological replicate. (J) Overlap of poised promoters enriched for H3K27me3 in CEDT over CE and TN over CETN adipocytes. (K) Genome browser track of scaled H3K4me3 and H3K27me3 signals at promoters of *Ebf2* and *Tbx15*. Scaling was done per hPTM. (L) PCA plot of H3K4me1 quantified in adipocyte specific enhancers which were obtained by merging white and beige/brown enhancers. Each dot corresponds to an individual biological replicate. (M) Overlap of enhancers enriched in CE adipocytes over CEDT and in CETN adipocytes over TN. (N) Overlap of enhancers enriched in CEDT adipocytes over CE and in TN adipocytes over CETN. CEDT *n* = 2, CE ingAT *n* = 4, TN ingAT *n* = 3, CETN ingAT *n* = 4. Fisher’s exact test with Benjamini-Hochberg method for correction of multiple testing was used for GSEA (D, e). Significance of associations of MOFA factor scores to variables was tested using fitted ANOVAs using Benjamini-Hochberg procedure for correction for multiple testing (G). (For interpretation of the references to colour in this figure legend, the reader is referred to the Web version of this article.)

Intrigued by our observation of potential poising of the *Ucp1* promoter in beige adipocytes, we wondered whether white adipocytes or UCP1- adipocytes might repress or poise *Ucp1* through H3K27me3 deposition. To determine if adipocytes that can beige (UCP1+) are inherently different from those that do not respond to cold exposure with *Ucp1* expression, a question that has not been fully resolved, we used AdipoCre x NuTRAP x Ucp1DTR mice. In these mice, diphtheria toxin administration targets UCP1+ cells due to the *Ucp1* promoter-driven expression of the diphtheria toxin receptor [[33], [34], [35]], enabling the specific ablation of UCP1+ cells. We injected diphtheria toxin subcutaneously after cold exposure into the flank of these mice (CEDT) (Figure 4A) to effectively target the ingAT (Fig. S5A) and verified the ablation of UCP1+ cells via Western Blot (Fig. S5A) for each ingAT sample. We then isolated adipocyte nuclei from these ingAT samples and performed CUT&Tag and examined H3K4me3 levels at the *Ucp1* locus in adipocytes from cold-exposed mice. As expected, CEDT adipocytes showed reduced H3K4me3 deposition at the *Ucp1* promoter compared to adipocytes from saline-injected control CEDT mice and CE beige adipocytes (Figure 4B). Although we cannot confirm complete ablation, our CUT&Tag data from CEDT adipocytes likely represents non-beige adipocytes collected shortly after CE.

To explore the differences between non-beige and beige adipocytes, as well as thermoneutral white adipocytes (TN) isolated from AdipoCre x NuTRAP mice housed at 30 °C from birth on (Figure 4A), we analysed H3K4me3 deposition at promoters. PCA of H3K4me3 deposition revealed that CE beige adipocytes were distinct from CETN beige, CEDT and TN white adipocytes along PC1. Non-beige adipocytes clustered with warmed beige adipocytes suggesting similarities in H3K4me3-marked promoters between these groups (Figure 4C). Indeed, only 30 promoters were differentially marked by H3K4me3 between CEDT and CETN adipocytes (Table S5). GSEA of differentially enriched promoters between CEDT and CE adipocytes revealed that CEDT adipocytes, similar to CETN beige adipocytes (Figure 3D), exhibited H3K4me3 enrichment at promoters related to IL-6 production and signalling (Figure 4D), including genes such as *Cd74* (Figure 4F). CE adipocytes, as expected, displayed H3K4me3 enrichment at promoters of thermogenic regulators, including *Prdm16* (Figure 4D,F).

We next asked what differentiates CEDT from TN adipocytes and compared H3K4me3 promoter deposition. Interestingly, only 106 promoters showed differentially enrichment (Table S6). In TN adipocytes, these promoters were associated with genes involved in ECM related pathways, such as *Postn* (Figure 4E–F). Conversely, in CEDT adipocytes, promoters of genes related to DNA repair, such as *Wdr19* (Figure 4E–F), were enriched for H3K4me3, suggesting potential apoptotic signal transduction persisting after diphtheria toxin injection. However, the low number of promoters that were enriched in CEDT adipocytes might have skewed this analysis.

Since H3K4me3 did not strongly differentiate beige from non-beige adipocytes, we conducted MOFA using all datasets derived from adipocytes of ingAT (Fig. S5B). None of the MOFA factors could significantly separate white (TN and CEDT) from beige (CETN, CE, CERT) adipocytes. However, MOFA factor 2 showed a trend (*P* = 0.72) towards differentiating white from beige adipocytes, driven primarily by H3K27me3 and H3K4me1 (Figure 4G–H). Thus, we further analysed H3K27me3 at promoters (Figure 4I).

CEDT adipocytes showed differentially enrichment for H3K27me3 at 330 promoters, including *Ucp1* compared to CE beige adipocytes (Fig. S5C, Table S7), while TN adipocytes had 494 promoters enriched for H3K27me compared to CETN beige adipocytes (Fig. S5D, Table S8). Notably, TN adipocytes displayed repressive H3K27me3 enrichment at the *Ucp1* promoter, whereas in CETN beige adipocytes, *Ucp1* was poised (Figure 3H). These observations align with our observations of potential poising of thermogenic promoters upon rewarming in beige adipocytes, leading us to question whether white adipocytes repress or instead poised promoters. To address this, we analysed the co-presence of H3K4me3 at H3K27me3-enriched promoters. For both CEDT and TN adipocytes, these promoters were predominantly poised (90 % and 95 % respectively) rather than repressed (10 % and 5 % respectively) (Fig. S5C-D). Among these poised promoters, 105 overlapped between TN and CEDT (Figure 4J, Table S9). Remarkably, this group included *Ebf2*, *Tbx15* (Figure 4K), key driver genes involved in gene expression regulation of brown and beige adipocytes [[36], [37], [38]]. This suggests that non-beige white adipocytes are poised to induce signal transduction processes resulting in beigeing.

Roh et al. [7] demonstrated that beige adipocytes have a small set of enhancers distinct from warm white adipocytes. Building on this observation, we quantified beige/brown and white adipocyte specific enhancer sets, which we had compiled before (Figure 2E, Fig. S5E). We conducted a differential enrichment analysis of H3K4me1 in CEDT white adipocytes and CE beige adipocytes, and CETN and TN adipocytes respectively (Figure 4L). We found 410 beige/brown enhancers that were distinctly enriched in cold beige adipocytes compared to cold white adipocytes. Consistent with Roh et al. [7], we also found 103 enhancers that were enriched in CETN but not TN adipocytes (Figure 4M, Tables S10 and S11). However, there was minimal overlap between enhancers enriched in CETN and CE (Figure 4M). Among these were enhancers close to *Pik3r1*, *Sox6* and *Ucp1* (Fig. S6A-E). Further, when comparing enhancers enriched for H3K4me1 in CEDT or TN white adipocytes we found 185 enhancers that were enriched in both (Figure 4N, Table S12). These were related to EGFR and IL-6 signalling (Fig. S5F) and linked by proximity to genes such as Lyn and *Cd74* (Fig. S6D-E).

Overall, even under thermoneutral conditions, white adipocytes may poise promoters associated with thermogenesis-regulating genes [37,38], such as *Ebf2*, *Adra1a* or *Tbx15*. This could imply that all adipocytes from the ingAT have the potential to beige, provided the appropriate stimulus is applied to remove H3K27me3 from specific promoters. While mice housed at 22 °C during early adulthood certainly could have primed CEDT adipocytes for beigeing [39], at TN this seems less likely. Collectively, our MOFA and differential enrichment analysis results suggest that beige and white adipocytes in the ingAT do not represent distinct cell types but rather different cellular states, reflecting varying epigenetic states.

### 1.5 White adipocytes of epiAT have distinct enhancers and repress thermogenic genes

**Figure 5:**
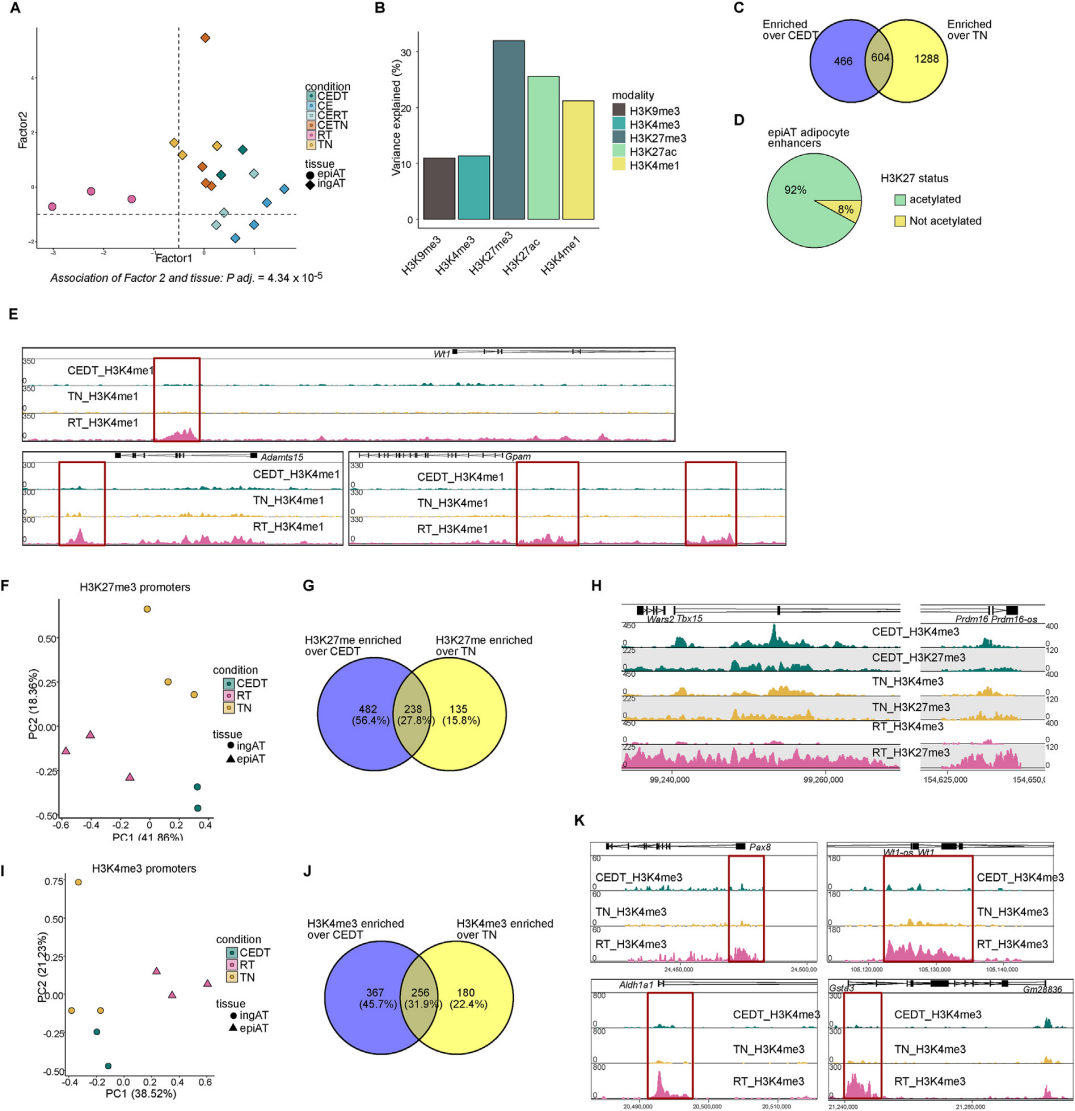
**White adipocytes of epiAT have distinct enhancers and repress thermogenic genes.** (A) MOFA factor plot showing the clustering along factors 1, 2 of labelled adipocytes from ingAT and epiAT of AdipoCre x NuTRAP mice. Association of MOFA factor 1 to tissue of origin is significant (*P* = 4.34 × 10^−5^). Each dot corresponds to one biological replicate. For each replicate all 5 hPTMs are represented in one dot. (B) Percentage of variance explained by MOFA factor 1 across 5 hPTMs. (C) Overlap of enhancers enriched in epiAT adipocytes over CEDT and over TN adipocytes. (D) H3K27ac status of enhancers from C. (E) Genome browser tracks of scaled H3K4me1 signal at enhancers close to *Adamts15*, *Gpam*, and *Wt1*. (F) PCA plot of H3K27me3 quantified in promoters of CE, CETN, CEDT and TN adipocyte samples. Each dot corresponds to an individual biological replicate. (G) Overlap of H3K27me3 enriched promoters in epiAT adipocytes over CEDT and TN adipocytes. (H) Genome browser tracks of scaled H3K27me3 and H3K4me3 signals at *Tbx15* and *Prdm16*. (I) PCA plot of H3K4me3 quantified in promoters of CE, CETN, CEDT and TN adipocyte samples. Each dot corresponds to an individual biological replicate. (J) Overlap of H3K4me3 enriched promoters in epiAT adipocytes over CEDT and TN adipocytes. (K) Genome browser track of scaled H3K4me3 at promoters of *Pax8*, *Wt1*, *Aldh1a1*, and *Gsta3*. CEDT *n* = 2, TN ingAT *n* = 3, RT epiAT *n* = 3, CE ingAT *n* = 4, CETN ingAT *n* = 4, CERT ingAT *n* = 3. Significance of associations of MOFA factor scores to variables was tested using fitted ANOVAs using Benjamini-Hochberg procedure for correction for multiple testing (A).

Given the similar epigenetic landscapes of beige and white ingAT adipocytes, we next explored whether white adipocytes from epiAT and ingAT exhibited as distinct differences as beige and brown adipocytes. We performed MOFA across all adipocyte datasets from ingAT and epiAT. Notably, H3K4me1 and H3K27 were key in explaining the variability in our data (Figure 5A–B). Particularly, H3K27me3 and, to a lesser extent, H3K27ac composed Factor 1 (Figure 5B), which significantly differentiated all ingAT from epiAT adipocytes (*P* = 4.34 × 10^−5^) (Figure 5A). We then compiled specific enhancers for white adipocytes from CEDT, TN and epiAT room temperature (RT) samples using ChromHMM and performed differential enrichment analysis between ingAT (both TN and CEDT) and epiAT white adipocytes. To derive epiAT-specific enhancer sets independent of the condition, we intersected our differential analysis results. We identified 604 enhancers enriched for H3K4me1 in epiAT adipocytes (Figure 5C, Table S13), of which 92 % were also marked by H3K27ac (Figure 5D). As expected, we found enhancers close to *Wt1* [40,41] in epiAT but not ingAT adipocytes (Figure 5E). Moreover, enhancers close to *Gpam* and *Adamts15* were enriched for H3K4me1 in epiAT adipocytes (Figure 5E). However, GSEA did not indicate that enhancers were related to specific pathways.

Based on our MOFA results we examined H3K27me3 enrichment at promoters. PCA revealed that H3K27me3 enrichment distinctly separated ingAT and epiAT adipocytes along PC1 (Figure 5F). We identified 238 promoters that are repressed by H3K27me3 in epiAT-derived adipocytes (Figure 5G, Table S14). Interestingly, key genes critical for thermogenesis [2,5,38] in brown and beige adipocytes, such as *Tbx15* and *Prdm16,* were marked by repressive H3K27me3 and depleted of H3K4me3, whereas in ingAT adipocytes these promoters are poised (*Tbx15*) or not repressed (*Prdm16*) (Figure 5H). Further differential analysis of H3K4me3 enrichment at promoters in epiAT adipocytes revealed consistent enrichment at 256 promoters (Figure 5I–J, Table S15). In line with our prior findings, H3K4me3 was also enriched at promoters of *Wt1, Pax8, Aldh1a1* and *Gsta3* (Figure 5K). GSEA revealed that the 256 epiAT adipocyte H3K4me3 enriched promoters were linked to biological processes such as fructose catabolism and morphogenesis of epithelial cells (Fig. S7).

In summary, white adipocytes derived from ingAT or epiAT exhibit distinct sets of enhancers. EpiAT adipocytes specifically repress genes involved in thermogenesis supporting the notion that epiAT and ingAT adipocytes are indeed of different origin as has been previously indicated in the literature [[40], [41], [42]].

## 2 Discussion

In this study, we present a detailed map of the healthy mouse adipocyte epigenome encompassing five hPTMs, across various AT depots, housing temperatures, and adipocyte phenotypes, thereby advancing prior work. We show that beige and brown adipocytes utilize distinct sets of enhancers, confirming their differing developmental origins [43]. Additionally, we observed that in warmed beige adipocytes promoters of thermogenic genes such as *Ucp1* are poised*,* supporting the notion that beige (and brown) adipocytes retain a “thermogenic memory”, which supports their ability to rapidly respond to thermogenic stimuli [7,44].

Furthermore, white adipocytes from epiAT and ingAT use different enhancers. Notably, mature adipocytes from the epiAT repress some thermogenic regulators at room temperature through H3K27me3 deposition, suggesting these cells are not inherently responsive to beigeing stimuli by expression of *Ucp1*. However, it has been shown that beta-adrenergic stimulation can induce the emergence of UCP1+ adipocytes within the epiAT [45], possibly originating from distinct progenitor cells rather than through the beigeing of existing mature adipocytes.

Several *in vivo* studies have suggested that beige adipocytes likely originate from two main pathways: the conversion of existing mature white adipocytes into beige adipocytes [34,35,45,46], and adipogenesis from potentially distinct precursor cells [45,[47], [48], [49], [50]]. Here, we used diphtheria toxin mediated ablation of beige adipocytes followed by isolation of remaining white adipocytes of the ingAT to show that these UCP1- white adipocytes are not inherently different from their UCP1+ positive beige counterparts. Notably, promoters of some thermogenic master regulators such as *Ebf2* and *Tbx15* are poised in non-beige adipocytes, but the adipocytes do not have distinct enhancers. This suggests that all ingAT adipocytes have the potential to beige given the correct stimulus and sufficient time, and likely share a common developmental origin and precursor. The ingAT adipocytes subtypes likely only reflect different cellular states.

In our epigenetic data, genes linked to other forms of thermogenesis such as creatine cycling [51,52] or calcium cycling [53] were not differentially enriched between beige and non-beigeing white adipocytes. This observation aligns with recent reports suggesting that adipocytes engaged in creatine cycling thermogenesis express only low levels of *Ucp1*, whereas classic beige adipocytes typically exhibit lower levels of *Alpl* [52,54]. Even low levels of *Ucp1* expression in cells using creatine cycling might be sufficient to induce recombination or DTR expression, which could explain why we do not observe differences in active mark deposition at genes like *Alpl*. It is also conceivable that, that epigenetically UCP1+ and other thermogenic adipocytes are not distinct from each other.

Given the complex interconnectedness of hPTMs and the mouse models used, there are inherent limitations that need to be acknowledged. Firstly, the GM mouse model Ucp1ERCre used to label beige and brown adipocytes was generated using a BAC spanning a ∼300 kb genomic region from *Tbc1d9* to *Clgn* [35]. Our analysis of H3K9me3 revealed that several copies or concatemers of this BAC likely integrated in heterochromatic regions and thus show H3K9me3 enrichment (Extended Data Fig. S6). It is important to note that H3k9me3 and H3K27me3 typically do not colocalise. Given that H3K9me3 was not enriched at the *Ucp1* gene body (Fig. S8), which in the BAC was replaced by the Cre recombinase, the H3K9me3 signals are unlikely to originate from the endogenous regulatory regions surrounding *Ucp1*. While this potential artifact does not specifically affect the comparison of beige to brown adipocytes – since both BAT and ingAT samples were always sampled from the same mouse, and CE and CETN mice were littermates – it cannot be entirely ruled out that the signals around *Ucp1* for other hPTMs may be impacted by the presence of multiple BAC copies. However, our findings regarding the poising of thermogenic genes or enhancer usage extend beyond the regulation of *Ucp1*. Therefore, our data likely reflects the true epigenetic landscape of beige and brown adipocytes. Nevertheless, our results underscore the need for careful epigenetic analysis of samples from genetically modified mice that were generated using BACs.

Overall, our study represents a significant step forward compared to previous epigenetic analyses, where samples were often pooled, and different pools were used to study different histone post-translational modifications (hPTMs). However, our paired datasets, while more comprehensive and complete, still do not finally prove whether promoters of thermogenic genes marked by both H3K4me3 and H3K27me3 are truly poised or if these signals arise from a heterogeneous population of adipocytes in which some cells repress a promoter while others activate it. To address this ambiguity, co-immunoprecipitation or novel single cell CUT&Tag [55] using nanobody-fused Tn5s experiments should be conducted.

In conclusion, our study offers an in-depth, unbiased, and comprehensive analysis of the mouse adipocyte epigenome. The enhancer and active promoters identified as specific to beige, white, or brown adipocytes not only shed light on the differential regulation among these cell types but may also aid in the design of novel genetically modified mice that could specifically target brown or beige adipocytes, or white adipocytes from different depots. Moreover, these findings can be used to identify new targets to enhance beigeing capacities, potentially through the use of tools such as epigenetic editing [56,57]. Lastly, the datasets generated in this study offer a valuable resource for further exploration of the mouse adipocyte epigenome and gene regulation, opening new avenues for research in metabolic regulation and obesity treatment.

## 3 Materials and methods

### 3.1 Data reporting

No statistical methods were used to predetermine sample size. The experiments were not randomized, and the investigators performing nuclei isolation of AT were not blinded to allocation during experiments and outcome assessment.

### 3.2 Mice

#### 3.2.1 Housing and lines

All mice were kept on a 12-h/12-h light/dark cycle and 20–60% (22 °C) humidity in individually ventilated cages in groups of two to five mice in a pathogen-free animal facility of ETH Zurich. The health of mice was monitored closely and any mouse exhibiting persisting clinical signs of ill-health or distress was excluded from this study. AdipoCre [58], Ucp1ERCre, Ucp1DTRGFP [33,35] and NuTRAP [10] mice were maintained on a C57BL/N background. Homozygous NuTRAP [10] and Cre lines mice were bred to generate AdipoCre x NuTRAP and Ucp1ERCre x NuTRAP mice. AdipoCre x NuTRAP mice were bred with homozygous Ucp1DTRGFP mice to generate AdipoCre x NuTRAP x Ucp1DTRGFP mice. All mice had *ad libitum* access to water and a standard chow diet (diet no. 3437, Provimi Kliba SA). Experiments were started with 8-week-old male mice. All animal experiments were approved by the cantonal veterinary office Zurich.

#### 3.2.2 Cold exposure

Mice were cold exposed at 8 °C after a gradual temperature decrease over 8 h in groups of 3 in their home cage for 1 week [34].

#### 3.2.3 Thermoneutral housing

Mice were housed at 30 °C in their home cages.

#### 3.2.4 Tamoxifen application

Ucp1ERCre x NuTRAP were gavaged three times with 1 mg tamoxifen dissolved in corn oil during cold exposure.

#### 3.2.5 Diphtheria toxin injections

100 ng diphtheria toxin (Sigma Aldrich, #322326; Lot #3461122) dissolved in 0.9% NaCl solution (saline) was injected subcutaneously in the flank or scruff region of the animal three times per day in 6-hour intervals for two consecutive days after cold exposure [33]. Control mice were injected with saline.

### 3.3 Western Blot

Snap frozen ingAT tissues were homogenized in ice-cold RIPA buffer containing 1x cOmplete™ EDTA-free Protease Inhibitor (Roche, #5056489001). After three centrifugations steps at 4 °C, protein lysates were quantified using a detergent-compatible colorimetric protein assay (BioRad, # 5000112). 20 ug protein was loaded onto an 12% SDS-PAGE gel for separation and blotted onto a nitrocellulose membrane (BioRad, #1620112). Membranes were incubated in TBST-BSA (5% w/v) for 1 h, then incubated with primary antibodies (anti-Ucp1, 1:1000 (abcam, #ab10983); and anti-γ-tubulin, 1:1000 (Sigma–Aldrich, #T6557)) in TBST-BSA over night at 4 °C. Membranes were washed three in TBST and incubated with secondary antibodies (StarBright Blue 700 anti-mouse, 1:5000 (BioRad, #12004158) and Alexa Fluor 488, anti-rabbit, 1:1000 (ThermoFisher Scientific, #A-11034). Membranes were imaged with a ChemiDoc MP imager (BioRad).

### 3.4 CUT&Tag

#### 3.4.1 Isolation of nuclei and pulldown

Nuclei were isolated from snap frozen adipose tissue depots in ice cold Nuclei Extraction Buffer (Miltenyi, #130-128-024) supplemented with 10 mM sodium butyrate and 1x cOmplete™ EDTA-free Protease Inhibitor (Roche, #5056489001) using the gentleMACS™ Octo Dissociator (Miltenyi, #130-096-427) using C-tubes (Miltenyi, #130-093-237). All centrifugation steps were carried out in a to 4 °C cooled swinging bucket centrifuge with settings at 500 g for 5 min. Nuclei were subsequently filtered through a 50 μm cell strainer (Sysmex, #04-0042-2317) and washed two times in PBS-BSA (1% w/v) containing protease inhibitor and sodium butyrate. Nuclei were centrifuged and subsequently bound to pre-blocked Dynabeads MyOne Streptavidin C1 beads (ThermoFisher, #65002) for 30 min at 4 °C followed by 3 washes with PBS-BSA (1% w/v) on a magnetic stand.

#### 3.4.2 Library preparation

CUT&Tag was performed as previously described with minor adjustments [9,59]. All buffers were supplemented with 1 x cOmplete EDTA-free Protease inhibitor and 10 mM sodium butyrate. Briefly, nuclei bound to beads were aliquoted into 96-well LoBind plates (Eppendorf, #0030129547) and incubated with primary antibodies – anti-H3K4me3 (abcam, #ab8580), anti-H3K27me3 (Cell Signaling Technology, #C36B11), anti-H3K27ac (abcam, #ab4729), anti-H3K4me1 (abcam, #ab8895), anti-H3K9me3 (abcam, #ab8898) – overnight at 4 °C. All antibodies were diluted 1:100. With the plate on a magnet the primary antibody solution was removed, the beads were resuspended in secondary antibody solution (guinea pig anti-rabbit IgG (antibodies-online gmbh, #ABIN101961); 1:200 dilution) and incubated at room temperature for 30 min while rocking. pA–Tn5 was bound to antibodies, transposition performed at 37 °C and stopped using TAPS-Wash solution. Nuclei were lysed and pA–Tn5 decrosslinked using SDS-release solution. PCR was performed using Kapa HiFi plus dNTPs (Roche Diagnostics, #07958846001) with the following PCR settings: 72 °C 5 min, 98 °C 30 s, 5 cycles of 98 °C 10 s, 63 °C 30 s, 72 °C final extension for 1 min, hold at 4 °C. After 5 cycles the PCR was put on hold at 4 °C and 10% of the reaction was transferred into a 384 well plate and mixed with KAPA SYBR 2x (qPCR) and primers and qPCR was run for 20 cycles to determine the number of PCR cycles to be added for each library [60]. Libraries were cleaned using SPRI beads using a 1.3 ratio, eluted in nuclease free water and pooled equimolecularily after library quantification using the KAPA library quantification kit (Roche Diagnostics, #079602). Libraries were sequenced at PE150 on a NovaSeq 6000 at Novogene at a targeted read depth of 3G per library.

### 3.5 Sequencing data processing

#### 3.5.1 Quality control and peak annotation

Quality control of CUT&Tag and data and generation of bedgraph files was performed as described previously [9,61]. Peaks were called from CUT&Tag-seq libraries on individual bedgraph files using SEACR [62] v1.3 in stringent mode with a peak calling threshold of 0.01. Peaks overlapping with mouse blacklist regions [63] were filtered out. For H3K9me3 analysis the region encompassing the BAC that was used to generate the Ucp1ERCre mice [35] was included in the blacklist due to the observed H3K9me3 signals stemming from the likely presence of BAC concatemers in heterochromatin regions (Fig. S8).

Called peaks were annotated using the R package ChIPSeeker [64]. Peak fold enrichment against genomic features was calculated using the formula:

Σ (bp overlap) ∗ genome_size /[Σ (bp PTM peak)∗Σ (bp genomic feature)].

Genomic features tracks were downloaded from ENCODE using the R package annotatr [65]. Visual QC of bam files was performed with Seqmonk [66]. Called peaks were combined to generate a union peak list and quantified using the R package chromVAR [67] v1.16 generating a raw peak count matrix.

#### 3.5.2 MOFA

MOFA2 [24,25] was run to identify the driving variation source across all conditions using all data modalities. For each modality, the top 3000 variable peaks between all samples were selected using the R package DESeq2 [68] and used as input to train the MOFA model. The trained MOFA model represented data variability in terms of five latent factors, which were further explored and visualized. Associations of MOFA factors with covariates were tested using the MOFACellulaR package [26].

#### 3.5.3 Generation of enhancer tracks of adipocytes

Adipocyte chromatin states were identified using ChromHMM v1.22 [29] in concatenated mode with binned bam files (200 bp bins) from each condition combining all hPTMs for AdipoCre and Ucp1ERCre data respectively. After final model selection [61] with nine chromatin states and emission parameter calculation of hPTMs, chromatin state fold enrichment was performed against genomic features and ENCODE cCREs. Enhancer states were selected based on genomic localization and hPTM enrichment. Regions in the genome that were marked by H3K4me1, H3K27ac but not enriched for H3K4me3 and were not promoters were identified as enhancers (Figure 2E, Fig. S5E). Subsequently, an enhancer track was generated per Cre driver.

#### 3.5.4 Differential enrichment analysis of hPTMs

##### 3.5.4.1 Promoters

Promoters were defined using the getPromoters function from ChIPSeeker with *TxDb.Mmusculus.UCSC.mm10.knownGene* as input and setting the TSSRegion to c(-2000, 2000). Peaks overlapping with promoters were extracted using the annotatePeak function from ChIPseeker [64] by selecting peaks annotated as promoters. For differential analysis our raw peak count matrix was filtered for these promoter regions and counts were aggregated at gene level. Differential analysis of the same hPTM between adipocytes stemming from different tissues was performed using the R package EdgeR [69] with FDR <0.05 and abs(log2FC) > 1 as cut-offs. For adipocytes derived from the same tissues nominal *p*-value <0.01 and abs(log2FC) > 1 was used. In the supplied supplementary files both nominal *p*-value and FDR are supplied.

##### 3.5.4.2 Enhancers

Our raw peak count matrix was filtered for enhancer regions defined by ChromHMM, and peaks around the TSS (+/− 2000 bp) were discarded. Linkage of putative enhancers to genes was done using the R package ChIPSeeker by selecting the closest gene (TSS or gene body) within 20,000 bp distance. Putative enhancers farther away than 20,000 from a TSS or gene body were not linked to any gene and were discarded from downstream GSEA.

For each hPTM, the raw filtered peak matrices were log normalized using the R package EdgeR and differential analysis of the same hPTM between adipocytes stemming from different tissues was performed using the R package EdgeR [69] with FDR <0.05 and abs(log2FC) > 1 as cut-offs. For adipocytes derived from the same tissues nominal *p*-value <0.01 and abs(log2FC) > 1 was used. In the supplied supplementary files DEGs with a log2FC > 0.5 are also supplied (with nominal *p*-value and FDR).

##### 3.5.4.3 Principal Component Analysis

Raw gene and promoter/enhancer specific peak count matrices were log normalized using the R package EdgeR. PCA of the normalized count matrices was performed using the prcomp function of R package stats v3.6.2.

##### 3.5.4.4 Gene set enrichment analysis

GSEA was performed using the R package enrichR [[70], [71], [72]].

#### 3.5.5 Visualization and statistical analysis

R v4.2 was used to analyse data and generate plots, and Seqmonk v1.48.1 was used to generate genome browser tracks using the Base Pair Quantitation Pipeline with correction for total count to largest store. Affinity Designer2 was used to adjust plots for clarity (*e*.*g*., colour schemes).

## CRediT authorship contribution statement

**Laura C. Hinte:** Writing – review & editing, Writing – original draft, Methodology, Investigation, Formal analysis, Data curation, Conceptualization. **Adhideb Ghosh:** Writing – review & editing, Software, Formal analysis, Data curation. **Daniel Castellano-Castillo:** Writing – review & editing, Methodology, Conceptualization. **Christian Wolfrum:** Writing – review & editing, Resources, Methodology, Funding acquisition. **Ferdinand von Meyenn:** Writing – review & editing, Supervision, Resources, Project administration, Funding acquisition, Formal analysis, Conceptualization.

## Funding

This work was supported by 10.13039/501100003006ETH Zurich core funding (FvM and CW), and a 10.13039/501100000781European Research Council Starting Grant (803491, BRITE to FvM).

## Declaration of competing interest

All authors declare they have no competing interests.

## Data Availability

All sequencing data are uploaded to GEO with accession number GSE262213.

## Appendix A Supplementary data

Multimedia component 1 Multimedia component 1

Multimedia component 2 **Supplementary Figure S1**. (A) Peak fold enrichment of called peaks from each CUT&Tag library for genomic features, scaled from −2 to 2. (B) Peak fold enrichment of called peaks from each CUT&Tag library for ENCODE cCREs, scaled from −2 to 2. H3K9me3 is not plotted for cCREs because it is not enriched or present in cCREs. cCREs, candidate cis-regulatory elements as defined by ENCODE. CTCF, not TSS-overlapping and with high DNase and CTCF signals only; DNase–H3K4me3, not TSS-overlapping and with high DNase and H3K4me3 signals only; dELS, TSS-distal with enhancer-like signatures; PLS, TSS-overlapping with promoter-like signatures; pELS, TSS-proximal with enhancer-like signatures. **Supplementary Figure S2**. Principal Component Analysis (PCA) plot of all quantified peaks per sample per hPTM. **Supplementary Figure S3**. MOFA heatmap displaying hierarchical clustering of all factor scores across all adipocyte samples. Upper colour panel shows the variance explained by each hPTM per factor (R2), and -logP results of ANOVA between factor scores and tissue. Lower colour panel indicates the tissue and Cre driver of each sample. Significance of associations of MOFA factor scores to covariates was tested using fitted ANOVAs using Benjamini-Hochberg procedure for correction for multiple testing. **Supplementary Figure S4**. (A) MOFA heatmap displaying hierarchical clustering of all factor scores across beige and brown adipocyte samples. Upper colour panel shows the variance explained by each hPTM per factor (R2), and -logP results of ANOVA between factor scores and tissue and condition. Lower colour panel indicates condition, tissue and Cre driver for each sample. Significance of associations of MOFA factor scores to covariates was tested using fitted ANOVAs using Benjamini-Hochberg procedure for correction for multiple testing. (A) H2K27ac status of genes linked to brown adipocyte specific enhancers across conditions. (C) H2K27ac status of genes linked to beige adipocyte specific enhancers across conditions. (D) Overlap of H3K9me3 enriched regions in brown or beige adipocytes during CE or (CE)TN. **Supplementary Figure S5**. (A) Western Blot of ingAT samples from CEDT mice injected subcutaneously with either saline or diphtheria toxin in the scruff or flank. ∗ denotes mice whose ingAT was used for CUT&Tag. (B) Poising status of H3K27me3 enriched promoters in CEDT adipocytes. (C) Poising status of H3K27me3 enriched promoters in TN adipocytes. (D) MOFA heatmap displaying hierarchical clustering of all factor scores across adipocyte samples from ingAT. Upper colour panel shows the variance explained by each hPTM per factor (R2), and -logP results of ANOVA between factor scores and tissue and condition. Lower colour panel indicates condition, tissue and Cre driver for each sample. Significance of associations of MOFA factor scores to covariates was tested using fitted ANOVAs using Benjamini-Hochberg procedure for correction for multiple testing. (E) ChromHMM analysis representation of the adipocyte hPTM profiles for AdipoCre adipocytes (left) by nine chromatin states. The colour scale corresponds to the emission parameter of each hPTM for each state. And fold enrichment of ChromHMM states for total genomic coverage and ENCODE cCREs scaled from -2 to 2 (right). State 9 is identified as enhancers. (F) Top (significant) pathway terms for genes linked to white adipocyte specific enhancers based on the Wikipathways database. Fisher’s exact test with Benjamini-Hochberg method for correction of multiple testing was used for GSEA. **Supplementary Figure S6**. (A-E) Genome browser track of scaled H3K4me1 signals of CE, CETN, CEDT and TN adipocytes in enhancers linked to *Pik3r1*, *Sox6*, *Ucp1*, *Lyn* and *Cd74*. **Supplementary Figure S7**. Top (significant) terms and -log of adjusted p-value for genes linked promoters enriched for H3K4me3 in epiAT adipocytes based on the GO Biological Process terms. Fisher’s exact test with Benjamini-Hochberg method for correction of multiple testing was used for GSEA. **Supplementary Figure S8**. Genome browser track of scaled H3K9me3 signals of adipocytes from all conditions of BAT, ingAT and epiAT. Red box denotes the region of the BAC that was used to create Ucp1ERCre mice. Enrichment of H3K9me3 is only apparent in adipocytes from Ucp1ERCre x NuTRAP mice. The absence of enrichment across the *Ucp1* gene body indicates that the H3K9me3 stems from the BAC rather than the endogenous locus. Multimedia component 2

## References

[1] Cohen P., Levy J.D., Zhang Y., Frontini A., Kolodin D.P., Svensson K.J. (2014). Ablation of PRDM16 and Beige Adipose causes metabolic dysfunction and a subcutaneous to visceral fat switch. Cell.

[2] Harms M.J., Ishibashi J., Wang W., Lim H.-W., Goyama S., Sato T. (2014). Prdm16 is required for the maintenance of brown adipocyte identity and function in adult mice. Cell Metab.

[3] Madsen J.G.S., Madsen M.S., Rauch A., Traynor S., Van Hauwaert E.L., Haakonsson A.K. (2020). Highly interconnected enhancer communities control lineage-determining genes in human mesenchymal stem cells. Nat Genet.

[4] Matsumura Y., Nakaki R., Inagaki T., Yoshida A., Kano Y., Kimura H. (2015). H3K4/H3K9me3 bivalent chromatin domains targeted by lineage-specific DNA methylation pauses adipocyte differentiation. Mol Cell.

[5] Ohno H., Shinoda K., Ohyama K., Sharp L.Z., Kajimura S. (2013). EHMT1 controls brown adipose cell fate and thermogenesis through the PRDM16 complex. Nature.

[6] Roh H.C., Kumari M., Taleb S., Tenen D., Jacobs C., Lyubetskaya A. (2020). Adipocytes fail to maintain cellular identity during obesity due to reduced PPARγ activity and elevated TGFβ-SMAD signaling. Mol Metabol.

[7] Roh H.C., Tsai L.T.Y., Shao M., Tenen D., Shen Y., Kumari M. (2018). Warming induces significant reprogramming of beige, but not brown, adipocyte cellular identity. Cell Metab.

[8] Siersbæk R., Rabiee A., Nielsen R., Sidoli S., Traynor S., Loft A. (2014). Transcription factor cooperativity in early adipogenic hotspots and super-enhancers. Cell Rep.

[9] Hinte L.C., Castellano-Castillo D., Ghosh A., Melrose K., Gasser E., Noé F. (2024). Adipose tissue retains an epigenetic memory of obesity after weight loss. Nature.

[10] Roh H.C., Tsai L.T.-Y., Lyubetskaya A., Tenen D., Kumari M., Rosen E.D. (2017). Simultaneous transcriptional and epigenomic profiling from specific cell types within heterogeneous tissues in vivo. Cell Rep.

[11] Kaya-Okur H.S., Wu S.J., Codomo C.A., Pledger E.S., Bryson T.D., Henikoff J.G. (2019). CUT&Tag for efficient epigenomic profiling of small samples and single cells. Nat Commun.

[12] Benayoun B.A., Pollina E.A., Ucar D., Mahmoudi S., Karra K., Wong E.D. (2014). H3K4me3 breadth is linked to cell identity and transcriptional consistency. Cell.

[13] Talbert P.B., Meers M.P., Henikoff S. (2019). Old cogs, new tricks: the evolution of gene expression in a chromatin context. Nat Rev Genet.

[14] Heintzman N.D., Hon G.C., Hawkins R.D., Kheradpour P., Stark A., Harp L.F. (2009). Histone modifications at human enhancers reflect global cell-type-specific gene expression. Nature.

[15] Creyghton M.P., Cheng A.W., Welstead G.G., Kooistra T., Carey B.W., Steine E.J. (2010). Histone H3K27ac separates active from poised enhancers and predicts developmental state. Proc Natl Acad Sci USA.

[16] Hou L., Xiong X., Park Y., Boix C., James B., Sun N. (2023). Multitissue H3K27ac profiling of GTEx samples links epigenomic variation to disease. Nat Genet.

[17] Millán-Zambrano G., Burton A., Bannister A.J., Schneider R. (2022). Histone post-translational modifications — cause and consequence of genome function. Nat Rev Genet.

[18] Schuettengruber B., Cavalli G. (2009). Recruitment of polycomb group complexes and their role in the dynamic regulation of cell fate choice. Development (Cambridge, England).

[19] Escobar T.M., Oksuz O., Saldaña-Meyer R., Descostes N., Bonasio R., Reinberg D. (2019). Active and repressed chromatin domains exhibit distinct nucleosome segregation during DNA replication. Cell.

[20] Riising E.M., Comet I., Leblanc B., Wu X., Johansen J.V., Helin K. (2014). Gene silencing triggers polycomb repressive complex 2 recruitment to CpG islands genome wide. Mol Cell.

[21] Voigt P., Tee W.-W., Reinberg D. (2013). A double take on bivalent promoters. Gene Dev.

[22] Allshire R.C., Madhani H.D. (2018). Ten principles of heterochromatin formation and function. Nat Rev Mol Cell Biol.

[23] Nicetto D., Zaret K.S. (2019). Role of H3K9me3 heterochromatin in cell identity establishment and maintenance. Curr Opin Genet Dev.

[24] Argelaguet R., Arnol D., Bredikhin D., Deloro Y., Velten B., Marioni J.C. (2020). MOFA+: a statistical framework for comprehensive integration of multi-modal single-cell data. Genome Biol.

[25] Argelaguet R., Velten B., Arnol D., Dietrich S., Zenz T., Marioni J.C. (2018). Multi-omics factor Analysis—A framework for unsupervised integration of multi-omics data sets. Mol Syst Biol.

[26] Ramirez Flores R.O., Lanzer J.D., Dimitrov D., Velten B., Saez-Rodriguez J. (2023). Multicellular factor analysis of single-cell data for a tissue-centric understanding of disease. eLife.

[27] Choi J., Lysakovskaia K., Stik G., Demel C., Söding J., Tian T.V. (2021). Evidence for additive and synergistic action of Mammalian enhancers during cell fate determination. eLife.

[28] Hnisz D., Abraham B.J., Lee T.I., Lau A., Saint-André V., Sigova A.A. (2013). Super-enhancers in the control of cell identity and disease. Cell.

[29] Ernst J., Kellis M. (2012). ChromHMM: automating chromatin-state discovery and characterization. Nat Methods.

[30] Moore J.E., Purcaro M.J., Pratt H.E., Epstein C.B., Shoresh N., Adrian J. (2020). Expanded encyclopaedias of DNA elements in the human and mouse genomes. Nature.

[31] Kajimura S., Spiegelman B.M., Seale P. (2015). Brown and beige fat: physiological roles beyond heat generation. Cell Metab.

[32] Cannon B., Nedergaard J. (2004). Brown adipose tissue: function and physiological significance. Physiol Rev.

[33] Challa T.D., Dapito D.H., Kulenkampff E., Kiehlmann E., Moser C., Straub L. (2020). A genetic model to study the contribution of brown and brite adipocytes to metabolism. Cell Rep.

[34] Moser C., Straub L.G., Rachamin Y., Dapito D.H., Kulenkampff E., Ding L. (2021). Quantification of adipocyte numbers following adipose tissue remodeling. Cell Rep.

[35] Rosenwald M., Perdikari A., Rülicke T., Wolfrum C. (2013). Bi-directional interconversion of brite and white adipocytes. Nat Cell Biol.

[36] Angueira A.R., Shapira S.N., Ishibashi J., Sampat S., Sostre-Colón J., Emmett M.J. (2020). Early B cell factor activity controls developmental and adaptive thermogenic gene programming in adipocytes. Cell Rep.

[37] Rajakumari S., Wu J., Ishibashi J., Lim H.-W., Giang A.-H., Won K.-J. (2013). EBF2 determines and maintains brown adipocyte identity. Cell Metab.

[38] Sun W., Zhao X., Wang Z., Chu Y., Mao L., Lin S. (2019). Tbx15 is required for adipocyte browning induced by adrenergic signaling pathway. Mol Metabol.

[39] Bruder J., Fromme T. (2022). Global adipose tissue remodeling during the first month of postnatal life in mice. Front Endocrinol.

[40] Kirschner K.M., Scholz H. (2022). WT1 in adipose tissue: from development to adult physiology. Front Cell Dev Biol.

[41] Chau Y.-Y., Bandiera R., Serrels A., Martínez-Estrada O.M., Qing W., Lee M. (2014). Visceral and subcutaneous fat have different origins and evidence supports a mesothelial source. Nat Cell Biol.

[42] Westcott G.P., Emont M.P., Li J., Jacobs C., Tsai L., Rosen E.D. (2021). Mesothelial cells are not a source of adipocytes in mice. Cell Rep.

[43] Harms M., Seale P. (2013). Brown and beige fat: development, function and therapeutic potential. Nat Med.

[44] Inoue S., Emmett M.J., Lim H.-W., Midha M., Richter H.J., Celwyn I.J. (2024). Short-term cold exposure induces persistent epigenomic memory in brown fat. Cell Metab.

[45] Lee Y.-H., Petkova A.P., Mottillo E.P., Granneman J.G. (2012). In vivo identification of bipotential adipocyte progenitors recruited by β3-Adrenoceptor activation and high-fat feeding. Cell Metab.

[46] Cinti S. (2009). Transdifferentiation properties of adipocytes in the adipose organ. Am J Physiol Endocrinol Metabol.

[47] Holman C.D., Sakers A.P., Calhoun R.P., Cheng L., Fein E.C., Jacobs C. (2023). Aging impairs cold-induced beige adipogenesis and adipocyte metabolic reprogramming. eLife.

[48] Oguri Y., Shinoda K., Kim H., Alba D.L., Bolus W.R., Wang Q. (2020). CD81 controls beige fat progenitor cell growth and energy balance via FAK signaling. Cell.

[49] Cattaneo P., Mukherjee D., Spinozzi S., Zhang L., Larcher V., Stallcup W.B. (2020). Parallel lineage-tracing studies establish fibroblasts as the prevailing in vivo adipocyte progenitor. Cell Rep.

[50] Park J., Shin S., Liu L., Jahan I., Ong S.-G., Xu P. (2021). Progenitor-like characteristics in a subgroup of UCP1+ cells within white adipose tissue. Dev Cell.

[51] Kazak L., Chouchani E.T., Jedrychowski M.P., Erickson B.K., Shinoda K., Cohen P. (2015). A creatine-driven substrate cycle enhances energy expenditure and thermogenesis in beige fat. Cell.

[52] Vargas-Castillo A., Sun Y., Smythers A.L., Grauvogel L., Dumesic P.A., Emont M.P. (2024). Development of a functional beige fat cell line uncovers independent subclasses of cells expressing UCP1 and the futile creatine cycle. Cell Metab.

[53] Ikeda K., Kang Q., Yoneshiro T., Camporez J.P., Maki H., Homma M. (2017). UCP1-independent signaling involving SERCA2b-mediated calcium cycling regulates beige fat thermogenesis and systemic glucose homeostasis. Nat Med.

[54] Wang T., Sharma A.K., Wu C., Maushart C.I., Ghosh A., Yang W. (2024). Single-nucleus transcriptomics identifies separate classes of UCP1 and futile cycle adipocytes. Cell Metab.

[55] Bartosovic M., Castelo-Branco G. (2023). Multimodal chromatin profiling using nanobody-based single-cell CUT&Tag. Nat Biotechnol.

[56] Policarpi C., Munafò M., Tsagkris S., Carlini V., Hackett J.A. (2024). Systematic epigenome editing captures the context-dependent instructive function of chromatin modifications. Nat Genet.

[57] Gemberling M.P., Siklenka K., Rodriguez E., Tonn-Eisinger K.R., Barrera A., Liu F. (2021). Transgenic mice for *in vivo* epigenome editing with CRISPR-Based systems. Nat Methods.

[58] Eguchi J., Wang X., Yu S., Kershaw E.E., Chiu P.C., Dushay J. (2011). Transcriptional control of adipose lipid handling by IRF4. Cell Metab.

[59] Henikoff S., Janssens D., S Kaya-Okur H., Henikoff J., Ahmad K. (2020).

[60] Buenrostro J.D., Giresi P.G., Zaba L.C., Chang H.Y., Greenleaf W.J. (2013). Transposition of native chromatin for fast and sensitive epigenomic profiling of open chromatin, DNA-binding proteins and nucleosome position. Nat Methods.

[61] Galle E., Wong C.-W., Ghosh A., Desgeorges T., Melrose K., Hinte L.C. (2022). H3K18 lactylation marks tissue-specific active enhancers. Genome Biol.

[62] Meers M.P., Tenenbaum D., Henikoff S. (2019). Peak calling by sparse enrichment analysis for CUT&RUN chromatin profiling. Epigenetics Chromatin.

[63] Amemiya H.M., Kundaje A., Boyle A.P. (2019). The ENCODE blacklist: identification of problematic regions of the genome. Sci Rep.

[64] Yu G., Wang L.-G., He Q.-Y. (2015). ChIPseeker: an R/Bioconductor package for ChIP peak annotation, comparison and visualization. Bioinformatics.

[65] Cavalcante R.G., Sartor M.A. (2017). Annotatr: genomic regions in context. Bioinformatics.

[66] Andrews S. (2022). https://www.bioinformatics.babraham.ac.uk/projects/seqmonk/.

[67] Schep A.N., Wu B., Buenrostro J.D., Greenleaf W.J. (2017). chromVAR: inferring transcription-factor-associated accessibility from single-cell epigenomic data. Nat Methods.

[68] Love M.I., Huber W., Anders S. (2014). Moderated estimation of fold change and dispersion for RNA-seq data with DESeq2. Genome Biol.

[69] Robinson M.D., McCarthy D.J., Smyth G.K. (2010). edgeR: a bioconductor package for differential expression analysis of digital gene expression data. Bioinformatics.

[70] Chen E.Y., Tan C.M., Kou Y., Duan Q., Wang Z., Meirelles G.V. (2013). Enrichr: interactive and collaborative HTML5 gene list enrichment analysis tool. BMC Bioinf.

[71] Kuleshov M.V., Jones M.R., Rouillard A.D., Fernandez N.F., Duan Q., Wang Z. (2016). Enrichr: a comprehensive gene set enrichment analysis web server 2016 update. Nucleic Acids Res.

[72] Xie Z., Bailey A., Kuleshov M.V., Clarke D.J.B., Evangelista J.E., Jenkins S.L. (2021). Gene set knowledge discovery with Enrichr. Curr Prot.

