# Supplementary material for: An epigenome atlas of mouse adipocytes
Source: Mol Metab. 2025 Jun 27;99:102197. doi: 10.1016/j.molmet.2025.102197 (PMC12274702; doi:10.1016/j.molmet.2025.102197)
Supplement: Multimedia component 2 — Supplementary Figure S1. (A) Peak fold enrichment of called peaks from each CUT&Tag library for genomic features, scaled from −2 to 2. (B) Peak fold enrichment of called peaks from each CUT&Tag library for ENCODE cCREs, scaled from −2 to 2. H3K9me3 is not plotted for cCREs because it is not enriched or present in cCREs. cCREs, candidate cis-regulatory elements as defined by ENCODE. CTCF, not TSS-overlapping and with high DNase and CTCF signals only; DNase–H3K4me3, not TSS-overlapping and with high DNase and H3K4me3 signals only; dELS, TSS-distal with enhancer-like signatures; PLS, TSS-overlapping with promoter-like signatures; pELS, TSS-proximal with enhancer-like signatures. Supplementary Figure S2. Principal Component Analysis (PCA) plot of all quantified peaks per sample per hPTM. Supplementary Figure S3. MOFA heatmap displaying hierarchical clustering of all factor scores across all adipocyte samples. Upper colour panel shows the variance explained by each hPTM per factor (R2), and -logP results of ANOVA between factor scores and tissue. Lower colour panel indicates the tissue and Cre driver of each sample. Significance of associations of MOFA factor scores to covariates was tested using fitted ANOVAs using Benjamini-Hochberg procedure for correction for multiple testing. Supplementary Figure S4. (A) MOFA heatmap displaying hierarchical clustering of all factor scores across beige and brown adipocyte samples. Upper colour panel shows the variance explained by each hPTM per factor (R2), and -logP results of ANOVA between factor scores and tissue and condition. Lower colour panel indicates condition, tissue and Cre driver for each sample. Significance of associations of MOFA factor scores to covariates was tested using fitted ANOVAs using Benjamini-Hochberg procedure for correction for multiple testing. (A) H2K27ac status of genes linked to brown adipocyte specific enhancers across conditions. (C) H2K27ac status of genes linked to beige adipocyte specific enhancer [file mmc2.pdf]

Figure S1

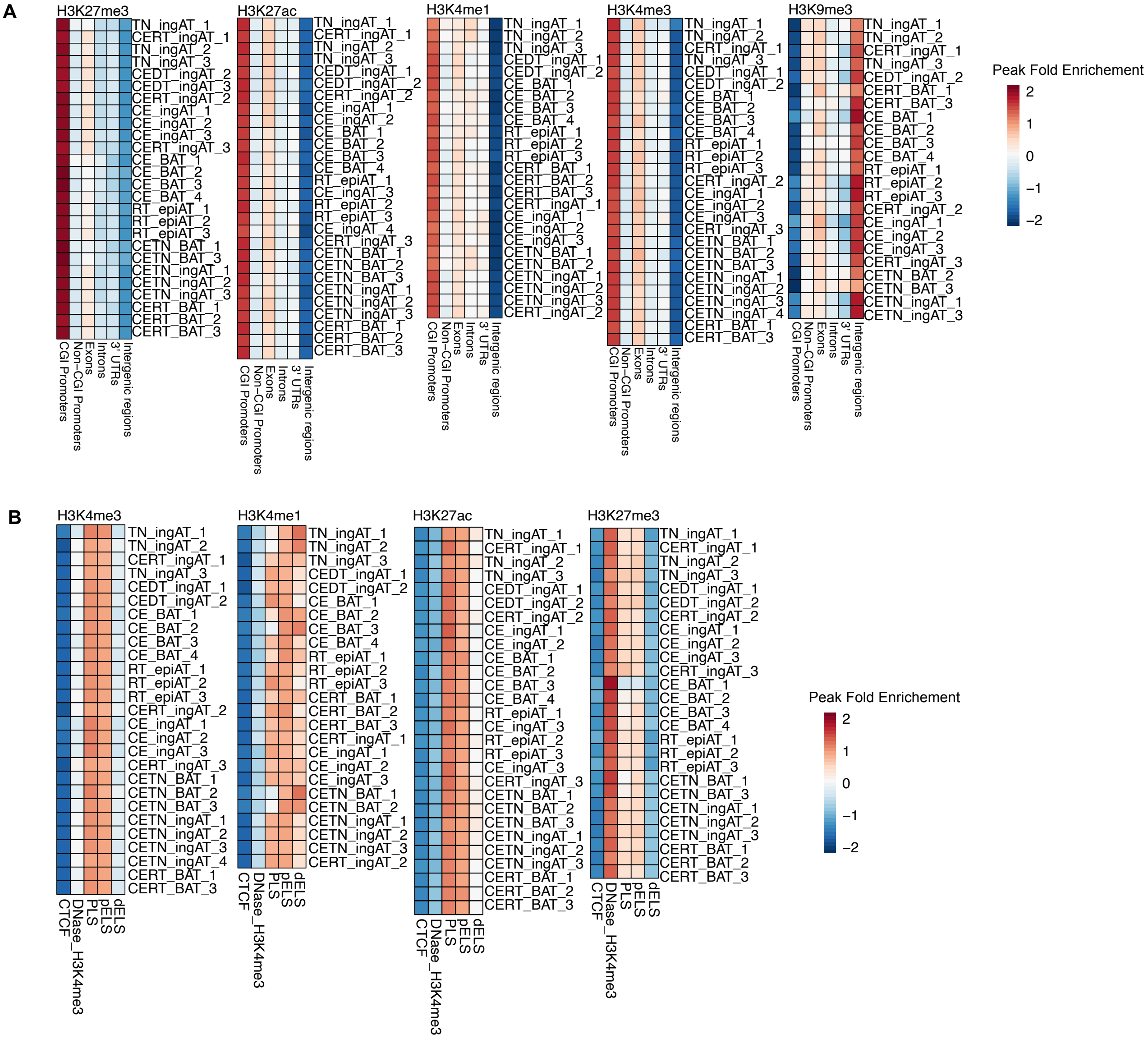
A
H3K27me3
H3K27ac
H3K4me1
H3K4me3
H3K9me3
CGI Promoters
Non−CGI Promoters
Exons
Introns
3' UTRs
Intergenic regions
Peak Fold Enrichement
2
1
0
−1
−2
B
H3K4me3
H3K4me1
H3K27ac
H3K27me3
CTCF
DNase_H3K4me3
PLS
pELS
dELS
Peak Fold Enrichement
2
1
0
−1
−2

Figure S2

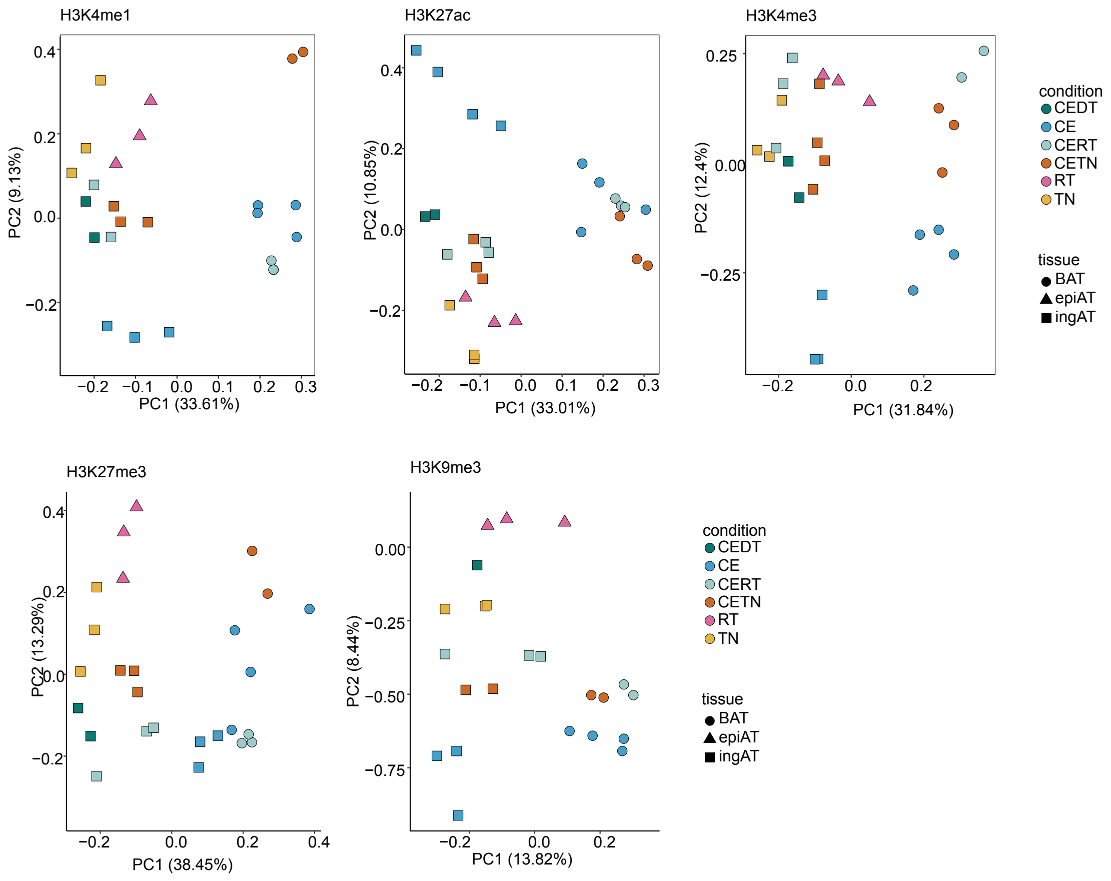

H3K4me1
PC1 (33.61%)
PC2 (9.13%)
H3K27ac
PC1 (33.01%)
PC2 (10.85%)
H3K4me3
PC1 (31.84%)
PC2 (12.4%)
H3K27me3
PC1 (38.45%)
PC2 (13.29%)
H3K9me3
PC1 (13.82%)
PC2 (8.44%)
condition
CEDT
CE
CERT
CETN
RT
TN
tissue
BAT
epiAT
ingAT

Figure S3

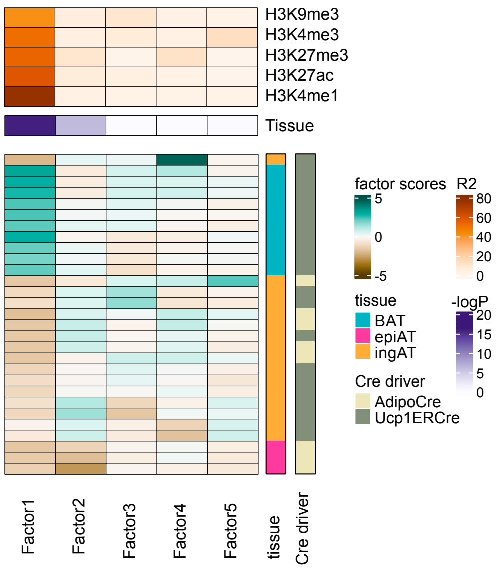
H3K9me3
H3K4me3
H3K27me3
H3K27ac
H3K4me1
Tissue
factor scores
5
0
-5
R2
80
60
40
20
0
tissue
BAT
epiAT
ingAT
-logP
20
15
10
5
0
Cre driver
AdipoCre
Ucp1ERCre
Factor1
Factor2
Factor3
Factor4
Factor5
tissue
Cre driver

Figure S4

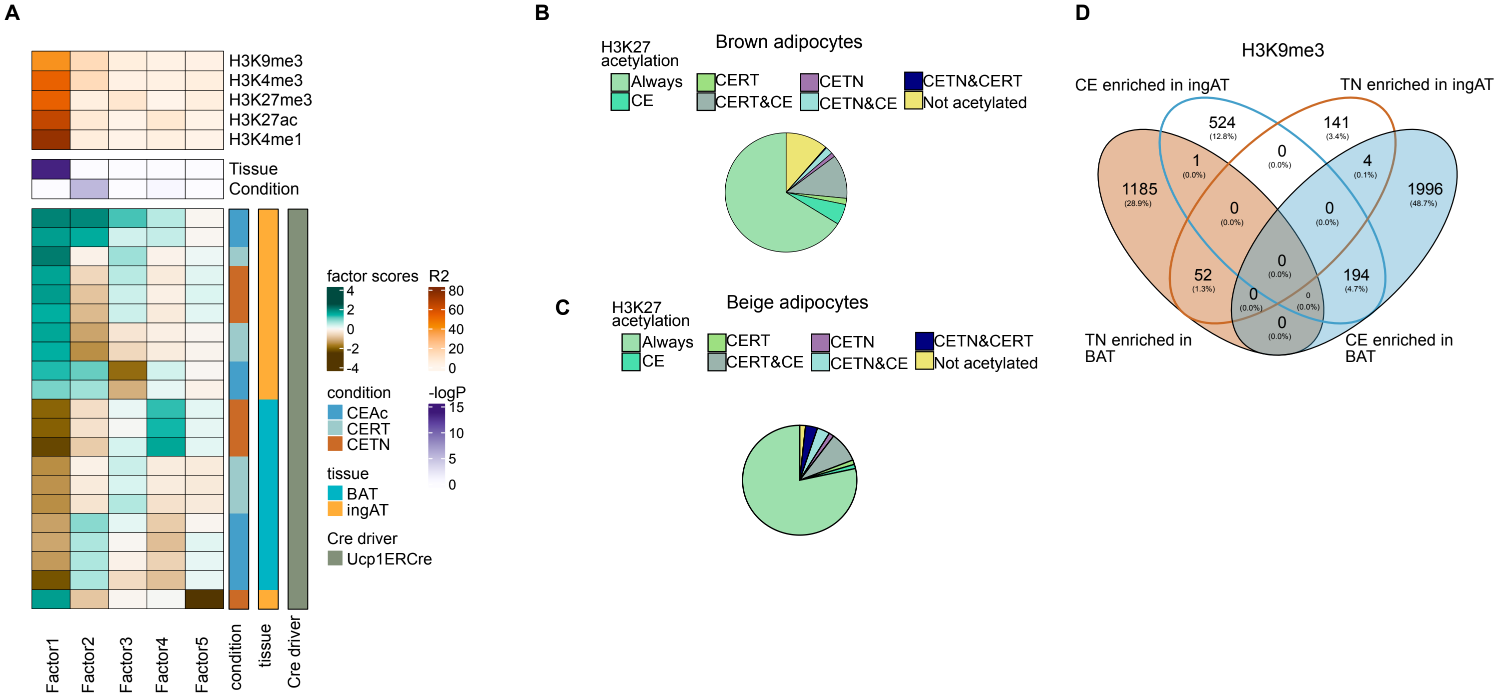
A
H3K9me3
H3K4me3
H3K27me3
H3K27ac
H3K4me1
Tissue
Condition
factor scores
R2
condition
CEAc
CERT
CETN
-logP
tissue
BAT
ingAT
Cre driver
Ucp1ERCre
Factor1
Factor2
Factor3
Factor4
Factor5
condition
tissue
Cre driver
B
Brown adipocytes
H3K27 acetylation
Always
CE
CERT
CERT&CE
CETN
CETN&CE
CETN&CERT
Not acetylated
C
Beige adipocytes
H3K27 acetylation
Always
CE
CERT
CERT&CE
CETN
CETN&CE
CETN&CERT
Not acetylated
D
H3K9me3
CE enriched in ingAT
TN enriched in ingAT
TN enriched in BAT
CE enriched in BAT
1185 (28.9%)
524 (12.8%)
141 (3.4%)
1996 (48.7%)
1 (0.0%)
4 (0.1%)
52 (1.3%)
194 (4.7%)

Figure S5

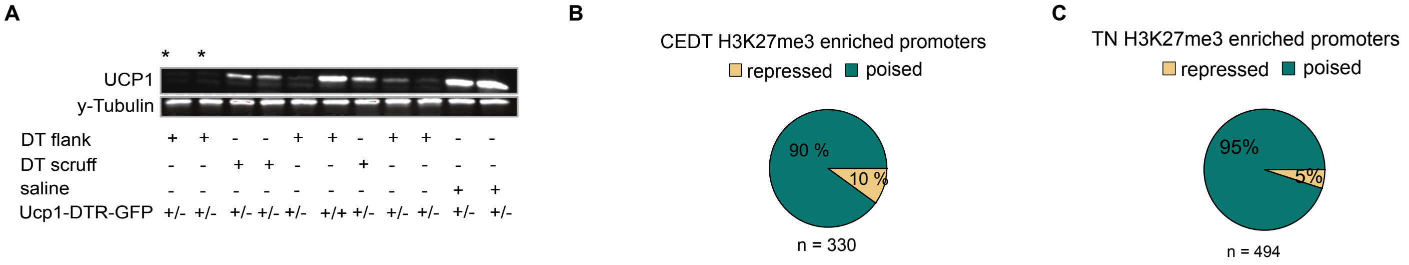

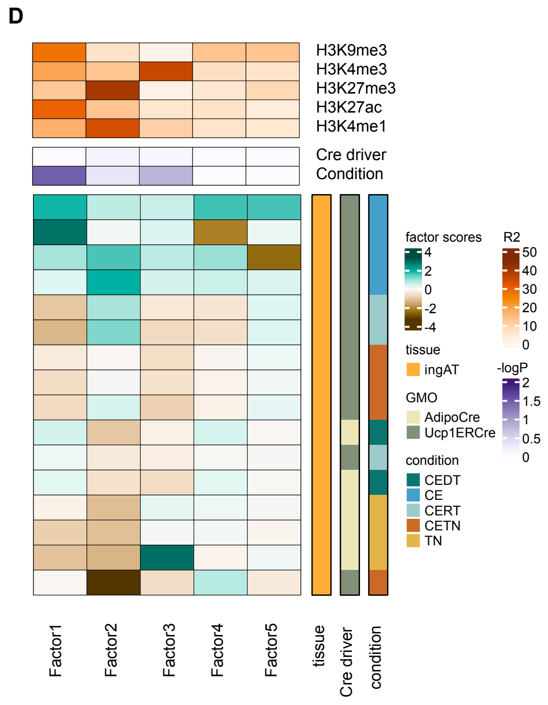

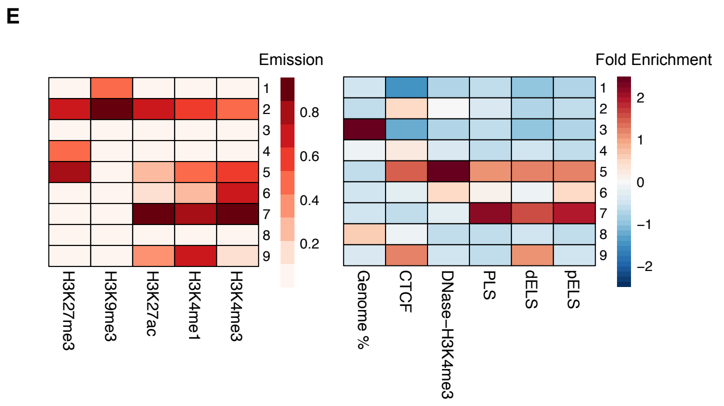

F

WikiPathways of genes linked to white enhancers	-logP	Gene Ratio
Kit Receptor Signaling Pathway WP407	1.958038	5/67
Calcium Regulation in the Cardiac Cell WP553	1.958038	7/147
GPCRs, Class B Secretin-like WP456	1.807115	5/69
EGFR1 Signaling Pathway WP572	1.772978	5/76
IL-6 signaling Pathway WP387	1.709006	3/22

Figure S6

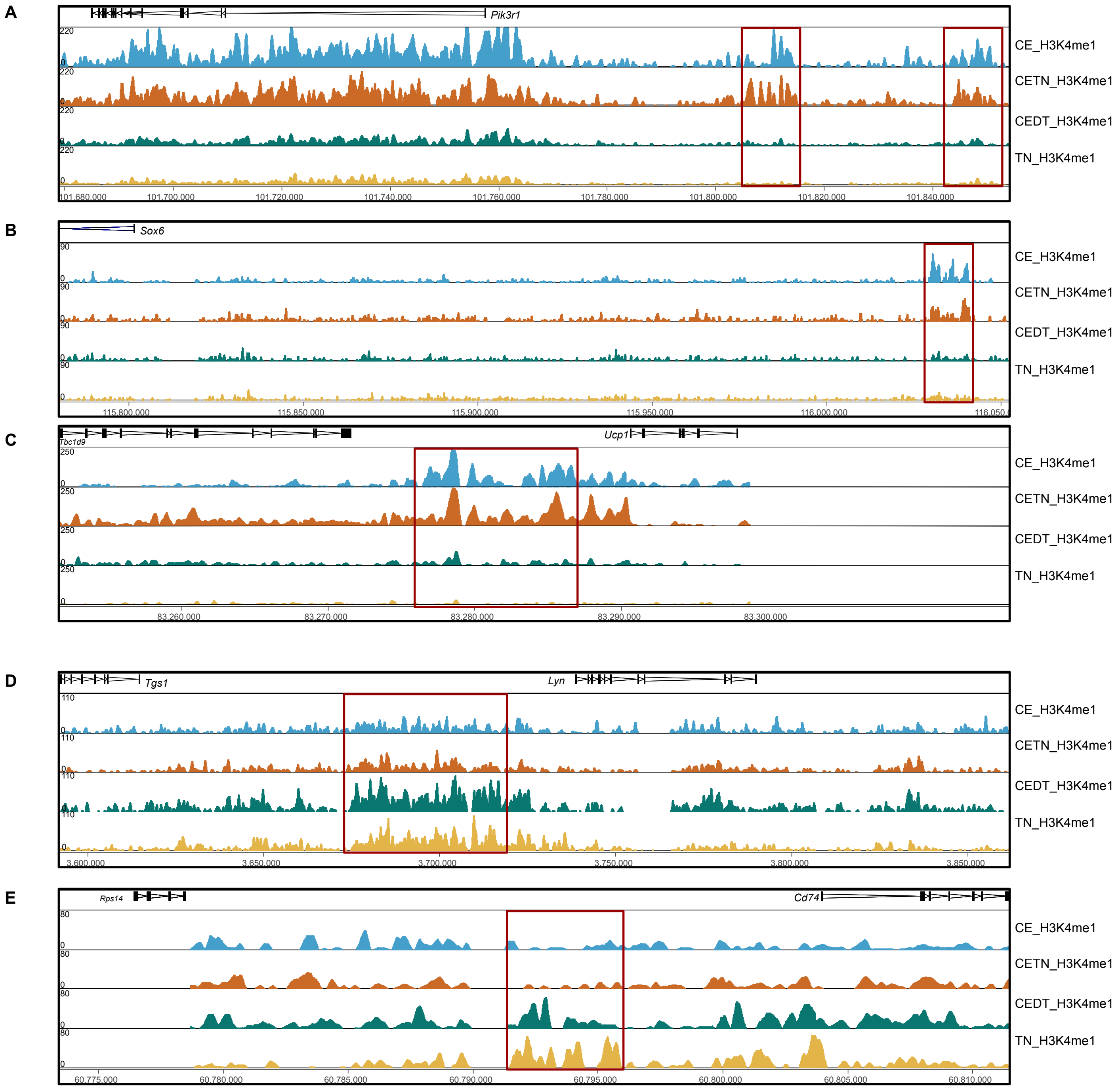
A
Pik3r1
CE_H3K4me1
CETN_H3K4me1
CEDT_H3K4me1
TN_H3K4me1
220
0
101,680,000
101,700,000
101,720,000
101,740,000
101,760,000
101,780,000
101,800,000
101,820,000
101,840,000
B
Sox6
CE_H3K4me1
CETN_H3K4me1
CEDT_H3K4me1
TN_H3K4me1
90
0
115,800,000
115,850,000
115,900,000
115,950,000
116,000,000
116,050,
C
Tbc1d9
Ucp1
CE_H3K4me1
CETN_H3K4me1
CEDT_H3K4me1
TN_H3K4me1
250
0
83,260,000
83,270,000
83,280,000
83,290,000
83,300,000
D
Tgs1
Lyn
CE_H3K4me1
CETN_H3K4me1
CEDT_H3K4me1
TN_H3K4me1
110
0
3,600,000
3,650,000
3,700,000
3,750,000
3,800,000
3,850,000
E
Rps14
Cd74
CE_H3K4me1
CETN_H3K4me1
CEDT_H3K4me1
TN_H3K4me1
80
0
60,775,000
60,780,000
60,785,000
60,790,000
60,795,000
60,800,000
60,805,000
60,810,000

Figure S7

GO BP	-logP	Gene Ratio
branching involved in ureteric bud morphogenesis (GO:0001658)	2.32541058	4/19
modified amino acid transport (GO:0072337)	1.61164108	3/8
branching morphogenesis of an epithelial tube (GO:0048754)	1.61164108	4/44
fructose catabolic process (GO:0006001)	1.50325648	2/5
fructose catabolic process to hydroxyacetone phosphate and glyceraldehyde-3-phosphate (GO:0061624)	1.50325648	2/5

Figure S8

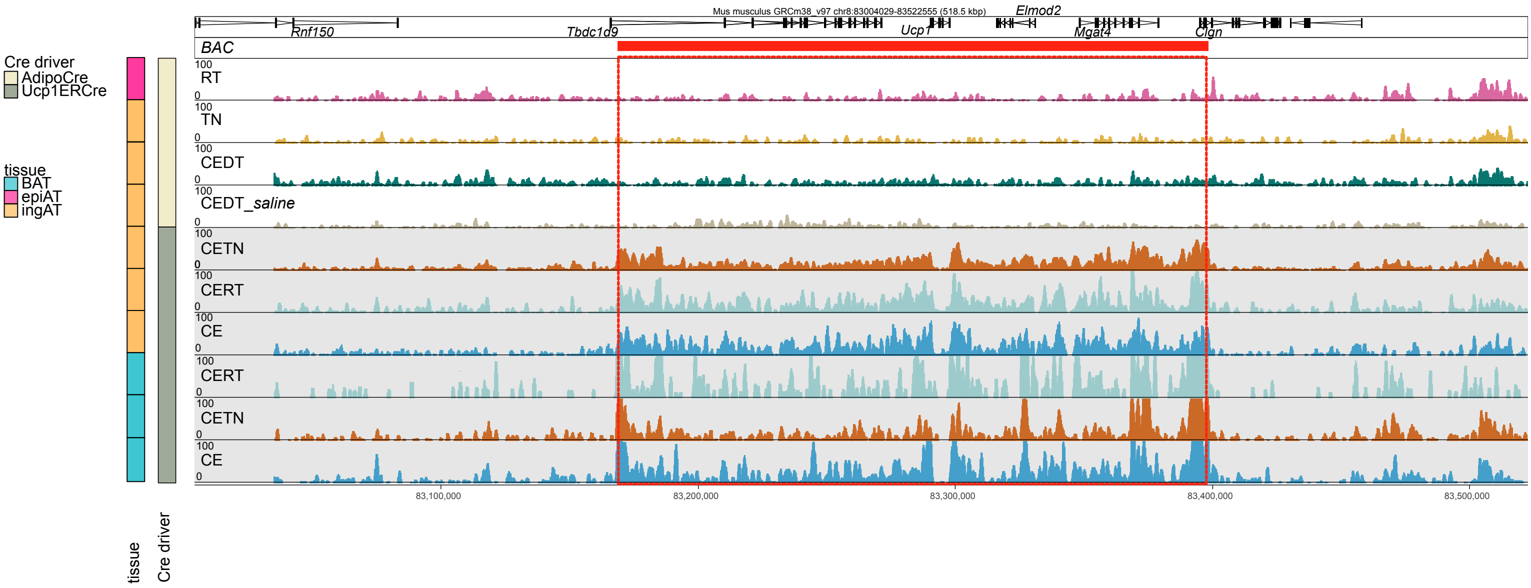

Cre driver
AdipoCre
Ucp1ERCre
tissue
BAT
epiAT
ingAT
Mus musculus GRCm38_v97 chr8:83004029-83522555 (518.5 kbp)
Elmod2
Rnf150
Tbdc1d9
Ucp1
Mgat4
Clgn
BAC
RT
TN
CEDT
CEDT_saline
CETN
CERT
CE
CERT
CETN
CE
83,100,000
83,200,000
83,300,000
83,400,000
83,500,000
tissue
Cre driver
